# Pattern preferences of DNA nucleotide motifs by polyamines putrescine^2+^, spermidine^3+^ and spermine^4+^

**DOI:** 10.1093/nar/gkz434

**Published:** 2019-05-22

**Authors:** Sergiy Perepelytsya, Jozef Uličný, Aatto Laaksonen, Francesca Mocci

**Affiliations:** 1 Bogolyubov Institute for Theoretical Physics of the National Academy of Sciences of Ukraine, 03143 Kyiv, Ukraine; 2 Department of Theoretical and Mathematical Physics, Kyiv Academic University, 03142 Kyiv, Ukraine; 3 Department of Biophysics, Institute of Physics, P. J. Šafárik University, 041 54 Košice, Slovakia; 4 State Key Laboratory of Materials-Oriented and Chemical Engineering, Nanjing Tech University, 210009 Nanjing, China; 5 Division of Physical Chemistry, Department of Materials and Environmental Chemistry, Arrhenius Laboratory, Stockholm University, 10691 Stockholm, Sweden; 6 Centre of Advanced Research in Bionanoconjugates and Biopolymers, Petru Poni Institute of Macromolecular Chemistry, Iasi, 700487, Romania; 7 Department of Chemical and Geological Sciences, University of Cagliari, I-09042 Monserrato, Italy

## Abstract

The interactions of natural polyamines (putrescine^2+^, spermidine^3+^ and spermine^4+^) with DNA double helix are studied to characterize their nucleotide sequence pattern preference. Atomistic Molecular Dynamics simulations have been carried out for three systems consisting of the same DNA fragment d(CGCGAATTCGCGAATTCGCG) with different polyamines. The results show that polyamine molecules are localized with well-recognized patterns along the double helix with different residence times. We observed a clear hierarchy in the residence times of the polyamines, with the longest residence time (ca 100ns) in the minor groove. The analysis of the sequence dependence shows that polyamine molecules prefer the A-tract regions of the minor groove – in its narrowest part. The preferable localization of putrescine^2+^, spermidine^3+^ and spermine^4+^ in the minor groove with A-tract motifs is correlated with modulation of the groove width by a specific nucleotide sequences. We did develop a theoretical model pointing to the electrostatic interactions as the main driving force in this phenomenon, making it even more prominent for polyamines with higher charges. The results of the study explain the specificity of polyamine interactions with A-tract region of the DNA double helix which is also observed in experiments.

## INTRODUCTION

The particular reversibly polymorphic 3D structural organization and the formation of the double helix DNA are determined by the surrounding environment of water and ions in several respects (1–3): hydrophobic bases of DNA are localized inside the double helix to minimize the contacts with water molecules, while the negatively charged sugar-phosphate backbone, faced to the exterior of the double helix is neutralized by positively charged counterions. Under natural conditions the counterions are metal ions (mainly Na^+^, K^+^ or Mg^2+^) or positively charged organic molecules like polyamines (putrescine^2+^, spermidine^3+^, spermine^4+^) (1). While the binding of monoatomic metal ions to DNA have been extensively studied and there is a vast amount of data featuring the character of their interaction with nucleic acids (4–24) many interaction features of the elongated polycations with DNA are still not clear. It is known that the interactions of the positively charged polyamine (PA) molecules with DNA have a significant biological effect including some emerging biotechnological applications (25–36). In this context, the sequence specific interactions of PAs with the DNA double helix warrant more thorough investigations.

The interaction of counterions with DNA double helix is governed by the long-range electrostatic forces inducing the condensation of counterions on the biopolyelectrolyte. The counterion condensation has been predicted by polyelectrolyte models (37,38) and confirmed by many experiments, in particular by the small angle X-ray scattering (SAXS) of DNA solutions (39–43). Condensed counterions are localized in a cloud around the macromolecule with a thickness of ∼7 Å, where they become an integral part of the dynamical structure of ion-hydration shell. Due to the spatial architecture of the double helix, characterized by inherently asymmetric minor and major groove running in parallel, and a backbone region bearing the largest electrostatic charge, the ion-hydration shell of DNA is far from being homogeneous system (3). The most important binding sites for counterions are the negatively charged phosphate groups of DNA backbone, exposed to the solution, and the electronegative pockets in the grooves of the double helix (1–10).

The localization of counterions around the double helix depends on the counterion properties, specific for each ion, such as charge, size, conformational flexibility and others. In the case of metallic counterions Na^+^, K^+^, Mg^2+^, commonly present in the cell medium, the interaction with DNA is determined by charge, size, and degree of hydration of the ions (24). The more strongly hydrated Na^+^ and Mg^2+^ ions are usually localized less deep inside the minor groove due to their strongly coordinated water molecules in their hydration shell (15,24), although some notable exceptions have received attention (6,8,9,11,12). K^+^ in turn can become more easily dehydrated and penetrate into the minor groove, interacting there with the atoms of the nucleotide bases (17–24). In the case of polyamines, their localization around the double helix is determined not only by the electrostatic charge but also by the length and conformational flexibility of the molecule, and the spatial distribution of the charges (44). The positively charged groups of a polycation molecule can bridge the negatively charged oxygen atoms of different phosphate groups. The phosphate groups may belong to the same or different strands of the double helix or even to different DNA molecules. Characterizing the interaction of polyamine molecules with the DNA double helix is much more complex than in the case of monatomic ions, and as a consequence much less is known concerning the interaction of these polycation with DNA.

DNA–polyamines interaction can largely affect the elastic properties of the double helix and their role in inducing aggregation and compaction of the macromolecule have been recognized since their early studies (45,46). Indeed, the condensation of DNA macromolecule is observed with counterions with charge ≥3, like spermidine^3+^ and spermine^4+^, and increasing the counterions concentration the effect becomes more prominent (46). In the eukaryotic cells, the concentration of polyamine molecules ranges between 0.88 and 1.58 mM (26), and this is much lower than the concentration of monovalent ions under physiological conditions 150 mM (1). Furthermore, the presence of polyamines in the system is proven to be indispensable for the dense packing of DNA macromolecule in chromatin (47) and viral capsids (48,49). In addition to the important role of polyamines in DNA condensation, it is also known that DNA–polyamine interactions induce changes in the secondary structure of the macromolecule, like *B*-*A* and *B*-*Z* conformational transitions of the double helix (44), that may be important with respect to different biological processes, where these conformations are involved (50,51). The polyamine molecules have also shown DNA radio-protective properties which are related to the facilitation of the recovery of DNA double stranded breaks induced by ionizing radiation (52). The observed effects of DNA–polyamine interactions emphasize the importance of a detailed study of the specificity of polyamine localization on the macromolecule to the sequence of DNA nucleotides.

The distribution of polyamines around the DNA double helix has been extensively studied, but earlier experimental studies and MD simulations did not indicate any prominent specificity to the nucleotide sequence (53–60). The experimental Raman spectra indicated that the interaction with the negatively charged phosphate groups are the strongest, and the sequence context of DNA was suggested playing only a secondary role in recognition (60). However other studies, experimental as well as computational, indicated the importance of the nucleotide sequence in DNA–polyamine interaction (25,30,36,61–66). The calculations of the energy of DNA fragment with spermine^4+^ using the method of atom-atom potential functions revealed lower energies of the complex in the case of poly(dA:dT) sequence compared to poly(dC:dG) (61). A combined MD and single-molecule fluorescence resonance energy transfer (FRET) study clearly revealed that in presence of spermine^4+^ the attractions between DNA macromolecules have sequence-dependent character, and this attraction is larger in the presence of AT-rich sequence compared to GC-rich (36). These results are also supported by recent calorimetric experiments, performed in presence of putrescine^2+^, spemidine^3+^ and spemine^4+^ on DNA macromolecules of different organisms containing different amount of CG nucleotide pairs (30); indeed, this study definitely shows that the energy of DNA–polyamine interaction is higher in the case of AT-rich DNA. Interactions of polyamines with A-tracts, a peculiar type of AT-rich sequence characterized by having at least four adjacent AT base pairs without any 5′-TpA-3′ step, were studied by employing uranyl photo-probing (63) and it did show a preferential binding of polyamines to A-tracts. However, the molecular details of DNA interactions with polyamines, in particular with A-tracts, and the driving force behind these interactions have not yet been addressed. To cover this gap, we present here a computational study combined with a theoretical model concerning the interactions of three different polyamines, putrescine^2+^, spermidine^3+^ and spermine^4+^, with a DNA sequence containing both A-tracts and alternating CG sequences. Extended MD simulations of DNA with counterions involving polyamines were performed, and the results concerning the experimentally observed sequence specificity are rationalized using a physical model.

The article is organized as follows. System set-up and simulation details are described in the second section. In the third section, the MD simulation results, showing the DNA minor groove in A-tract as the most preferable interaction site, are presented; the simple physical model describing the mechanisms of polyamine interactions with the DNA double helix is developed. The possible role of sequence specific localization of polyamine molecules in the minor groove of DNA double helix is discussed in the fourth section.

The obtained simulation results together with the physical model provide a molecular level understanding of the molecular interaction mechanisms behind the sequence specificity of the polyamine binding.

## MATERIALS AND METHODS

**Figure 1.**
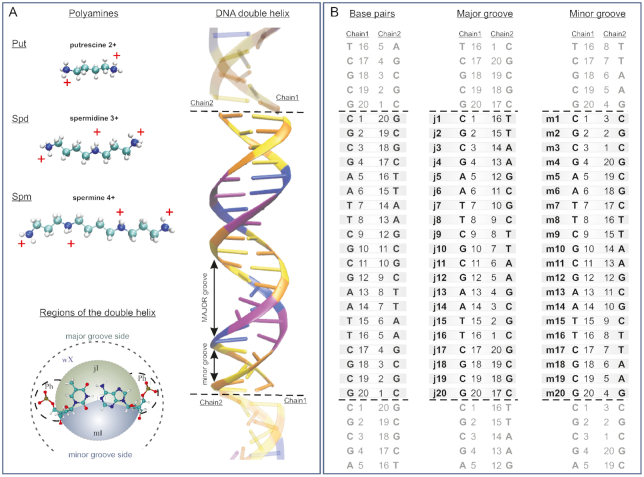
The simulated fragment of DNA with polyamines. (**A**) Schematic structure of polyamine molecules and the regions around the nucleotide pair of the double helix where the localization of the polyamine molecules is studied, defined according to ref. (17): mI and jI are the inner regions inside the minor and major groove, Ph—are the regions around the phosphate groups of the double helix backbone, wX is the outer regions of the minor and major groove sides around the double helix where the polyamine molecules are not constrained. Schematic structure of the simulated infinite DNA double helix is shown. Nucleotide colour scheme: Cytosine (orange), Guanine (yellow), Adenine (blue), Thymine (purple). The DNA images belonging to the upper and lower periodic box repetition are separated by dotted lines. (**B**) The notation of nucleotide pairs in the double helix; the numeration of the nucleotides in the major and minor grooves sides correspond to the shortest distance between phosphorus atoms on opposite strands of the double helix.

To perform the MD simulations three systems were constructed. Each system mimics an infinite macromolecule with repeating 20 base pair *B*-DNA fragment d(CGCGAATTCGCGAATTCGCG). The considered polynucleotide has two Drew-Dickerson sequences (67). The schematic structure of the DNA fragment and the notation of nucleotides in each strand of the double helix are shown in the Figure 1A and B. The particular notations of the regions of the DNA double helix where the polyamines may be located were adopted from the work (17). The DNA fragment was immersed in a water box with polyamine molecules of defined type: putrescine^2+^ (Put^2+^), spermidine^3+^ (Spd^3+^) or spermine^4+^ (Spm^4+^) (Figure 1A). In the present work, we neutralize the system using monovalent Na^+^ ions together with the polyamines putrescine^2+^, spermidine^3+^, and spermine^4+^, respectively (Supplementary Table S1 in Supplementary materials). The amount of Na^+^ counterions corresponds to 281, 270 and 288 mM, respectively. In this concentration range, the counterions may influence only the local structure of the double helix and in general DNA will keep the *B*-conformation (1,2). The detailed information about the simulated systems Put-DNA, Spd-DNA and Spm-DNA can be found in the Supplementary material (Table S1).

The computer simulations were performed using the NAMD software package (68). The parameter sets of CHARMM force field were used (69–71). For the nucleic acids and polyamines the CHARMM27 parameters were used. To control the quality of the nucleic acid force field, the system of DNA with spermidine^3+^ was simulated also using CHARMM36. The patch LKNA from CHARMM33 force field was used to link the ends of the DNA fragment with their images in the adjacent boxes, in order to mimic an infinite DNA double helix. The TIP3P model (72) of water molecules was used. The Beglov and Roux parameters were used for Na^+^ (73). The integration time step of 2 fs was used. The length of all bonds with hydrogen atoms was constrained using the SHAKE algorithm (74). The long-range electrostatic interactions were treated using particle mesh Ewald method (75). The switching and cut-off distances for the long-range interactions were set to 8 and 10 Å, respectively. The effect of using a longer cut-off radius for the long-range interactions was checked and the simulation results indicate that the characteristics of polyamine-DNA interactions do not change essentially with increased cut-off radii. The temperature was kept at 300 K using the Langevin thermostat for all heavy atoms (damping constant 5 ps^–1^). The oscillation time and damping time constants for the Langevin piston are 100 and 50 fs, respectively. The simulation length was of 1 μs for all the systems, except for the cut-off test which was performed on a 0.2 μs long simulation.

The parameters of polyamines are not directly implemented in the CHARMM force field. Therefore, in the present work the force field parameters for the polyamines were constructed using the parameters for the atoms of amino acids with amino groups available in CHARMM22 force field. This method of determination of the force constants for the simulations of DNA with polyamines was successfully used previously by Korolev *et al.* (53–56). The charges and types of the atoms of the polyamine molecules were assigned according to the scheme of CHARMM22 force field (Supplementary Figure S1 in Supplemental materials). The initial systems and the details of the simulation process are described in the Supplementary materials (Supplementary Figure S2 and Supplementary Table S2). VMD software package (76) was used for the system set up, analysis of simulation results, and visualization of the molecular structures.

### Equilibration

To analyze the stability and the convergence of the simulation the root mean square deviations (RMSD) were calculated for DNA and polyamine atoms along the entire trajectory (see Supplementary materials: Supplementary Figures S3–S5 for all systems calculated using CHARMM27 and Supplementary Figure S10 for the system with spermidine^3+^ calculated using CHARMM36). The results show that the RMSD fluctuates with time with respect to a mean value. The mean value in the case of DNA atoms is about 2.4 Å, low enough to consider the simulation stable. In the case of polyamine molecules, RMSD have the average value ∼1.3 Å revealing that the polyamine subsystem is equilibrated. During the time periods when the polyamine molecules are in the binding sites of DNA the fluctuations of the polyamine RMSD decrease significantly. Thus, the analysis of RMSD fluctuations shows that during the first 100 ns, DNA double helix and polyamine molecules become equilibrated. At the same time, the counterion sub-system may not yet be equilibrated due to its large mobility. Detailed long-trajectory MD simulation studies of DNA with counterions (17,19,22) indicate that the equilibration of counterions might require a rather long time, depending on the nucleotide sequence and on the DNA interaction site. For some DNA sequences, 100 ns is enough for equilibration of counterion sub-system, but in some cases calculation of much longer trajectory is necessary. Indeed, in the case of AAAA motive sequence in the minor groove even 1000 ns is not enough to reach the balance (19). The reason of such behaviour is not completely understood and is believed to be related to the specific interactions of counterions with the atoms in particular sites of the double helix and with the ability of the ions to constrain surrounding water molecules that essentially influences the DNA–counterion interactions (19). To check the equilibration of the system with respect to the interaction of polyamines with the DNA double helix, the distributions of the length of polyamine molecules were calculated over different time windows along the entire trajectory (Supplementary Figure S6 for all systems calculated using CHARMM27 and Supplementary Figure S11 for the system with spermidine^3+^ calculated using CHARMM36). The results show that after some time the shape of the distributions become similar for all of the polyamine molecules in each system: in the case of putrescine^2+^—after the first 200 ns, in the case of spermidine^3+^ and spermine^4+^—after about 400 ns. This behavior is connected to the residence time of the polyamine binding to DNA, which is shorter with putrescine^2+^ and allows all of the polyamines present in the system to sample both the bound and unbound states. In the case of spermidine^3+^, which has longer residence time at direct contact with DNA, in some time windows all of the three polyamines have the same average behavior, in some others they have different distribution. With the longest polyamine and highly charged spermine^4+^, the residence time is very long and both A-tracts host a polyamine for most of the trajectory. These results indicate that the systems are at the equilibrium during the production stage and that, as could be expected, the equilibration of DNA–polyamine system takes longer time in the case of longer and more highly charged polyamine molecules. In our simulations, we consider the first 200 ns of the simulation as part of the equilibration of the system; such period of time is more than enough for the equilibration of the structure of DNA double helix and polyamine molecules. The following 800 ns of simulation trajectory (from 201 to 1000 ns) can be considered representative of the equilibrium configurations of the systems, and were used for all of the analyses.

### Analysis details

The width of the groove was calculated as the minimal distance between two phosphate atoms of opposite strands of the macromolecule, as detailed in Figure 1B, subtracting 5.8 Å to the effective radii of the phosphates (1).

To analyze the conformational flexibility of the polyamines, the time-dependence of the distance between their terminal groups were calculated at each nanosecond of the simulation trajectory. Using the obtained results, the distribution of the distances was calculated for each polyamine molecule in each system for four time windows of the simulation trajectory.

To characterize the positioning of the polyamine molecule with respect to the atomic groups of the double helix, the distances from each atom of the polyamine backbone to the nearest atom of the phosphate group of DNA were calculated at every nanosecond from the simulation trajectory. These distances were averaged for each polyamine molecule of the system. To assign the regions (as defined in Figure 1A) where a polyamine is localized, the following criteria were adopted.

The polyamine molecule is localized inside the double helix (minor or major groove regions) if the average distance of the polyamine molecule to the center of the double helix was lower than 10 Å (the radius of DNA in *B*-form of the double helix). The atom N1 of the purine nucleotide was taken as the center of the double helix. The polyamine molecule was considered to be localized in the minor groove (region mI) or major groove (region jI) of the double helix if at least one heavy atom of the polyamine molecule was localized within 5 Å of nucleotide bases from the minor groove or major groove sides, respectively. In the grooves the following atoms are considered as the reference atoms: from the minor groove side - O2 of thymine and cytosine, C2 and N3 of adenine, N2 and N3 of guanine, O4′ and H4′ of deoxyribose; from the major groove side—C6, C5M, O4 of thymine, C6, C5, N4 of cytosine, C5, N7, and C8 of adenine and guanine, N6 of adenine, O6 of guanine. The polyamine molecule was attributed to be in the region near the phosphate groups (region Ph) if the polyamine molecule was not inside the grooves of the double helix and at least one heavy atom is within 7 Å of one of the oxygen atoms of the phosphate groups O1 or O2. If the polyamine molecule was outside the double helix and not constrained by the phosphate groups of the macromolecule it was considered to be in the free state (i.e. in the outside region wX). Thus, the space around the double helix where the polyamine molecule may be localized, is partitioned into four regions: minor and major grooves (mI- and jI- regions), phosphate groups of the double helix backbone (Ph-region), and the peripheral region outside of the double helix (wX region, see Figure 1A).

## RESULTS

### Molecular dynamics simulations of DNA–polyamine systems

**Figure 2.**
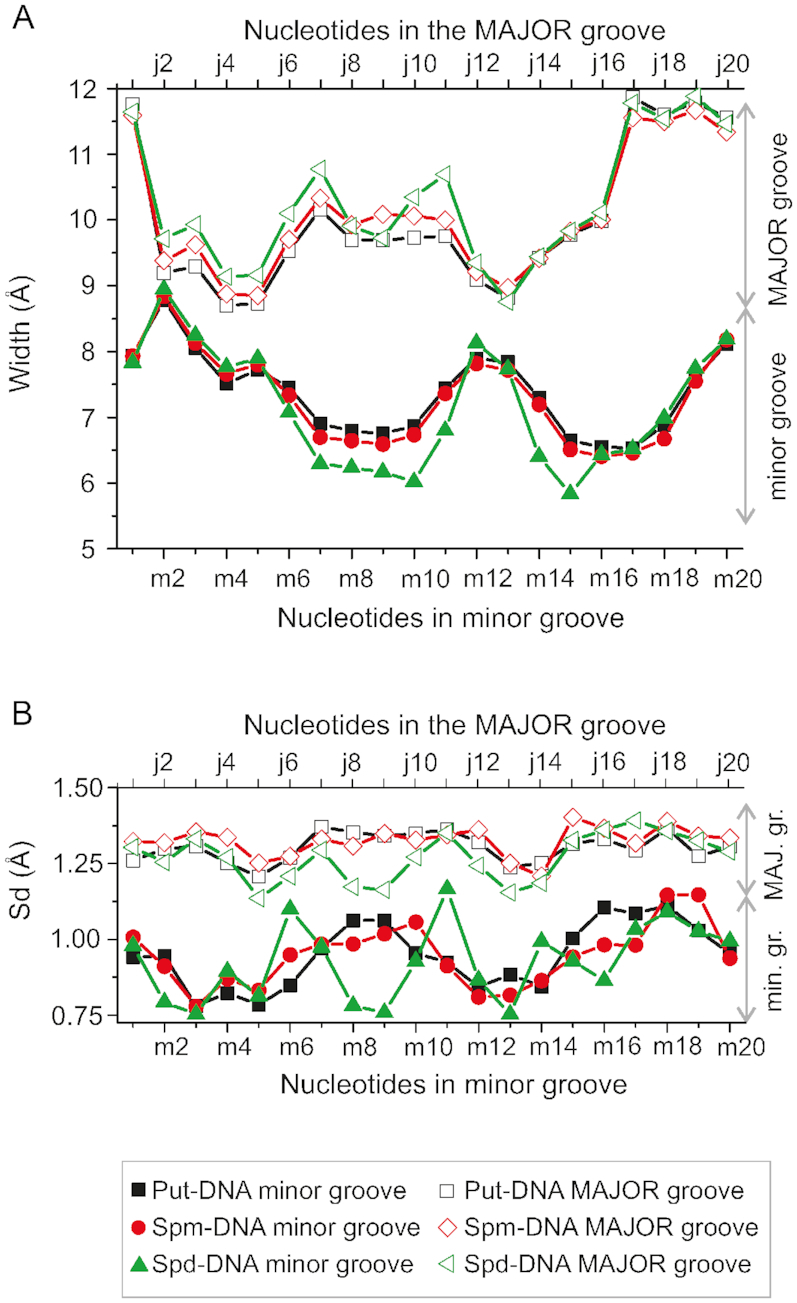
The dependencies of the average width (**A**) and standard deviations (**B**) of the minor and major grooves on nucleotide step. Black, red and green colors correspond to Put-DNA, Spd-DNA and Spm-DNA systems, respectively. To determine the van der Waals width of the groove the radius of the phosphates (5.8 Å) has been subtracted from the values of distances between phosphate groups. The numeration of the nucleotide steps from the minor and major groove sides are according to the Figure 1.

To characterize the interactions of the polyamine molecules with DNA, selected structural parameters of the double helix and of the polyamine were analyzed: the DNA grooves width, the end to end distances of the polyamines, the RMSD of each molecule with respect to the initial structure and the residence times in different regions of the double helix. The time dependencies of the widths of the grooves were calculated along the entire DNA chain, and were correlated to the nucleotide sequence, and their mean values are plotted in Figure 2A for all systems simulated using CHARMM27 and in Supplementary Figure S12A for the system with spermidine^3+^ calculated using CHARMM36.

As expected for *B*-DNA the width of the minor groove remains always smaller than the width of the major groove. The average values of their widths are 7.3 and 10.1 Å, respectively. For both the minor and the major grooves, the width is modulated by the sequence of nucleic bases. Using the notation introduced in Figure 1A, we can observe that the minor groove is narrowest (∼6.5 Å) in the regions of (m6÷m10) and (m14÷m18) nucleotides corresponding to the part of DNA with the (AATT):(TTAA) nucleotide motif. The minor groove is widest (∼8 Å) in the regions (m1÷m4), (m19÷m20) and (m11÷m13) that correspond to (CGCG):(CGCG) nucleotide motif. The major groove is narrowest (∼14.5 Å) in the regions (j2÷j5) and (j10÷j14), while the widest part of the major groove is in the regions (j6÷j12) and (j12÷j20). Furthermore, an anti-correlation of the width of minor and major grooves is clearly present: the regions with the narrowest minor groove have the widest major groove, and vice versa (Figure 2A). The fluctuations of the grooves are characterized by standard deviation of the width with the mean value as reference. Standard deviations obtained for the simulation trajectory at each nucleotide step show that there is a difference for the minor and major grooves (Figure 2B for all systems calculated using CHARMM27 and Supplementary Figure S12B for system with spermidine^3+^ calculated using CHARMM36). Thus, the amplitudes of the width changes of the grooves are higher for the major groove than for the minor groove.

As far as the conformational flexibility of the polyamines is concerned, the distributions of the end-to-end distance (DEED) can give useful information, and are plotted in Supplementary Figures S6 and S11. In the case of putrescine^2+^, the distributions have two prominent maxima characterizing the bent and stretched states of the molecule, while in the case of spermidine^3+^ and spermine^4+^ molecules the distribution is multimodal, with several overlapping maxima characterizing the bent and stretched conformations. For spermine^4+^, the strong maximum at 15.33 Å, corresponding to the stretched conformation indicates that this is still slightly bent due to the contact with DNA. In our simulations, we observe that polyamines in the bulk are in a bent conformation, which is preferable due to conformational entropy, while the stretched conformations are observed for the polyamine molecules interacting with the DNA double helix dominantly via electrostatics. In the case of putrescine^2+^ the DEED is the same for all considered simulation windows, while in the case of the spermidine^3+^ and spermine^4+^ the DEED of the different polyamine molecules become similar only in the second half of the simulation trajectory. The time required for the convergence of the distribution of the length of polyamine molecule, is related to the residence times of polyamines in DNA binding sites and increases with the polyamine length and charge. The conformational flexibility of the polyamine molecules is one of the determinative features for their localization in the binding sites.

**Figure 3.**
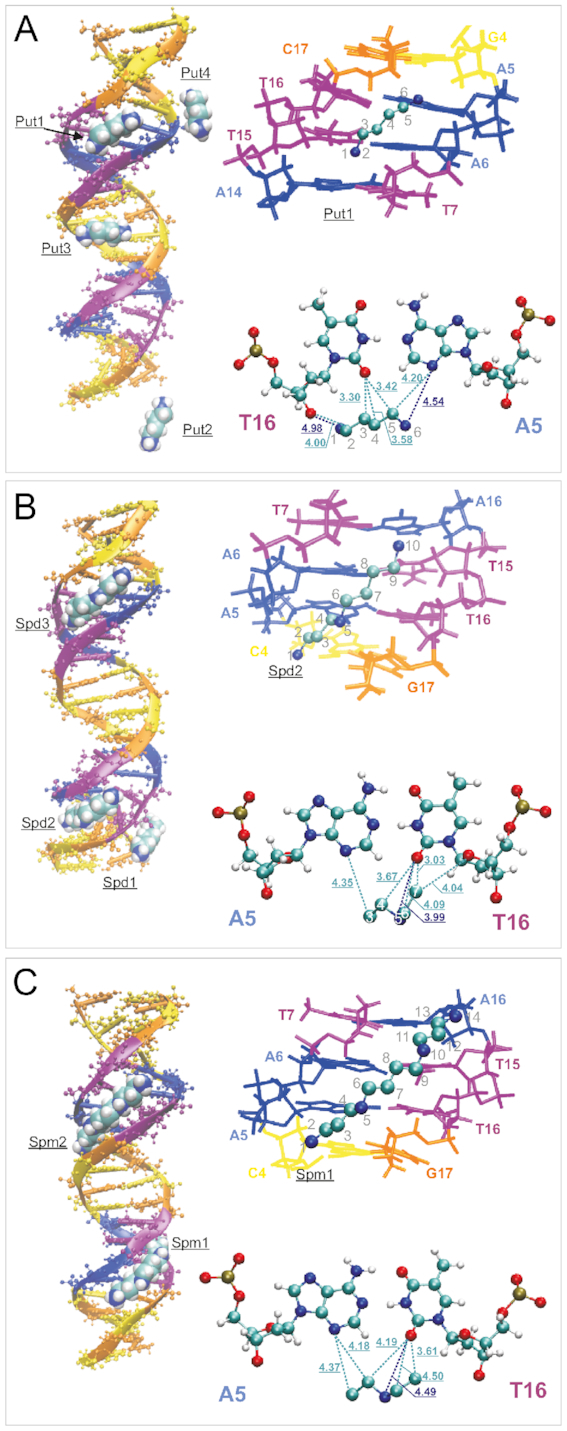
Selected snapshots of the systems revealing the typical binding sites of polyamine molecules with DNA double helix. On the left are the characteristic positions of the polyamine molecules shown. On the right at the top the details of the positioning of the selected polyamine molecule in the minor groove are shown. On the right at the bottom the distances from some polyamine atoms to the nearest atoms of the nucleotide pair are shown (Å). The atoms in the backbone of polyamine molecule are numerated. (**A**) Put-DNA system. Put1, Put2, Put3, Put4 are the labels of the putrescine^2+^ molecules; (**B**) Spd-DNA system. Spd1, Spd2, Spd3 are the labels of the spermidine^3+^ molecules; (**C**) Spm-DNA system. Spm1, Spm2 are the labels of the spermine^4+^ molecules. Nucleotides color scheme: Cytosine (orange), Guanine (yellow), Adenine (blue), Thymine (purple).

An analysis of the binding sites of polyamine molecules with DNA, through visual examination of the dynamics of the system coordinates, shows that polyamine interactions with DNA tend to be localized in the minor groove of the double helix, and that the binding frequently starts with a direct contact with the phosphate groups, thereafter the polyamine crawls into the minor groove. The snapshots of the complexes with polyamine molecule in the DNA minor groove show that putrescine^2+^, spermidine^3+^, and spermine^4+^ are all similarly located (Figure 3). The polyamines form direct contacts mostly with O2 atoms of purine and N3 atoms of pyrimidine nucleotide bases (the distance between the atoms of the polyamine backbone and the atoms of nucleotide fluctuates ∼4 Å). The distance to the other atoms of DNA in the minor groove is larger but still not so large to host a water molecule. Thus, the polyamines in the minor groove replace the water molecules thereby disturbing the hydration shell of DNA double helix.

The trajectories of the average distances of each polyamine molecule to the nearest phosphate group (Supplementary Figures S7A, S8A, S9A, S13A) allow localizing the polyamine molecules with respect to the DNA double helix. The total (${\tau _{{\rm{tot}}}}$), maximal (${\tau _{\max }}$), and mean (${\tau _{{\rm{mean}}}}$) residence times of the polyamine in the four regions defined in Figure 1A, are summarized in Table 1 and Supplementary Table S4. The residence times clearly depend on the type of polyamine molecule and increase with the charge of the molecule. In the minor groove, the both total and maximal residence times are the longest. The values of ${\tau _{{\rm{tot}}}}$ and ${\tau _{\max }}$are generally of the same magnitude among different polyamines in a given region around DNA. The characteristic residence times in the major groove are substantially shorter than those in the minor groove, being one order of magnitude smaller. Such difference points out a higher mobility of polyamine molecules in the major groove than in the minor groove. The localization of polyamines near the phosphate groups are characterized by the lowest values of residence times indicating a high degree of molecular mobility in this region outside the grooves.

**Table 1.** Residence times of polyamine molecules in different regions around DNA double helix: ${\tau _{{\rm{tot}}}}$, ${\tau _{\max }}$, and ${\tau _{{\rm{mean}}}}$ – the total, maximal, and mean residence times of localization (in ns). The designation of regions around DNA macromolecule: mI – minor groove, jI – major groove, Ph –backbone, wX – outside region (Figure 1A). *N*_0_ is the number of heavy atoms (C, N) in the polyamine chain

	Localization of polyamine molecule around DNA double helix											
	mI-region			jI- region			Ph- region			wX-region		
Polyamine	${\tau _{{\rm{tot}}}}$	${\tau _{\max }}$	${\tau _{{\rm{mean}}}}$	${\tau _{{\rm{tot}}}}$	${\tau _{\max }}$	${\tau _{{\rm{mean}}}}$	${\tau _{{\rm{tot}}}}$	${\tau _{\max }}$	${\tau _{{\rm{mean}}}}$	${\tau _{{\rm{tot}}}}$	${\tau _{\max }}$	${\tau _{{\rm{mean}}}}$
Purescine^2+^ (*N*_0_= 6)												
Put1	268.2	28.6	2.2	51.0	9.0	0.1	9.2	0.4	0.0	471.6	30.0	1.5
Put2	205.8	19.8	0.9	48,6	5.0	0.1	12.2	0.2	0.0	533.4	16.4	1.3
Put3	150.2	21.4	1.1	64,8	5.2	0.1	9.0	0.2	0.0	576.0	24.0	2.0
Put4	246.0	42.0	2.0	39,4	5.2	0.0	9.0	0.2	0.0	505.6	20.0	1.7
Aver.	217.6	28.0	1.6	51.0	6.1	0.1	9.9	0.3	0.0	521.7	22.6	1.6
Spermidine^3+^ (*N*_0_= 10)												
Spd1	127.8	16.8	0.3	88.0	6.6	0.1	17.0	0.4	0.0	567.2	39.0	2.0
Spd2	415.8	69.0	10.9	52.0	8.4	0.1	15.2	0.4	0.0	317	22.6	1.2
Spd3	566.2	81.0	13.3	5.6	1.0	0.0	11.6	0.4	0.0	216.6	17.2	0.9
Aver.	369.9	55.6	8.2	48.5	5.3	0.1	14.6	0.4	0.0	366.9	26.3	1.4
Spermine^4+^ (*N*_0_= 14)												
Spm1	635.8	71.8	17.73	0.0	0.0	0.0	11.0	0.4	0.0	153.2	21.6	1.0
Spm2	523.8	71.2	17.20	12.0	2.8	0.0	9.2	0.4	0.0	255.0	16.2	1.0
Aver.	580.0	71.5	17.47	6.0	1.4	0.0	10.0	0.4	0.0	204.1	18.9	1.0

The analysis of the occupancy of polyamine molecules reveals a hierarchy of the residence times in the three binding regions on the DNA surface:

(1) \begin{equation*}{\tau _{mI}} >{\tau _{jI}} > {\tau _{Ph}}.\end{equation*}

where ${\tau _{mI}}$, ${\tau _{jI}}$ and ${\tau _{Ph}}$ are the characteristic residence times of the polyamine molecules in the minor groove, major groove, and in the regions near the phosphate groups, respectively. The only exception to this trend is observed for one of the spermine^4+^ (Spm1) which spend most of the time of the simulation in the minor groove, and therefore does not interact with the major groove.

To analyze the dependence of polyamine localization on the base pairs sequence, the nucleotides closest to the polyamine molecule were identified at each saved trajectory step and the time-dependence of the nearest numbered nucleotide was plotted for each polyamine molecule at each nanosecond of the simulation trajectory (Supplementary Figures S7B, S8B, S9B and S13B). The summary of the characteristic sequence specificity of polyamine positioning on DNA is reported in the Table 2. The binding sites are shown as the sequence of nucleotides in the first and the second chains between which the polyamine molecule is positioned. Using these data, the regions where the polyamine molecule is located and the position of nucleotide were determined and marked using the notation presented in Figure 1A.

**Table 2.** The position of the nucleotides of the first and the second chain of the double helix where the polyamine molecules are localized most frequently. The numeration of the nucleotide bases and the corresponding regions are shown on the Figure 1b

System	Polyamine	Region
Put-DNA	Put1	mI: m14-m17
	Put2	mI: m16-m18
	Put3	mI: m7-m10
	Put4	mI: m14-m16
Spd-DNA	Spd1	jI: j10-j13
	Spd2	mI: m6-m10
	Spd3	mI: m15-m18
Spm-DNA	Spm1	mI: m7-m9
	Spm2	mI: m14-m16

**Figure 4.**
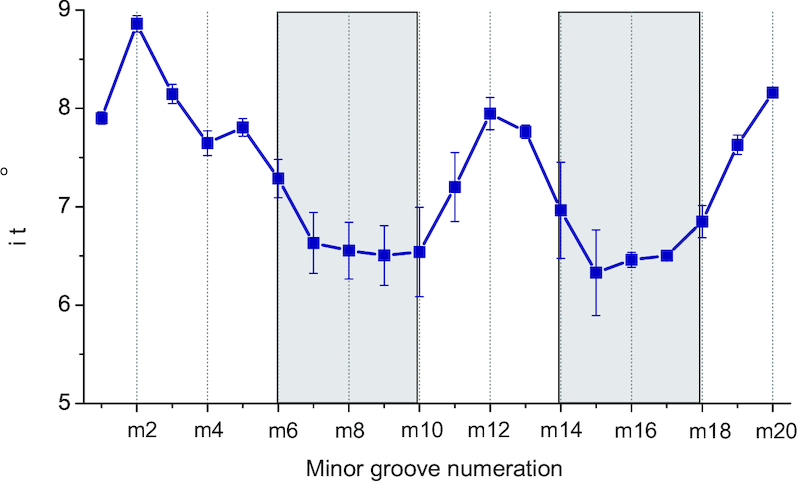
The dependence of the width of the minor groove on nucleotide sequence averaged over all of the simulated systems. The most probable localization of the polyamines is shown by grey bands.

The results in Table 2 show that there is a clear sequence specificity of polyamine binding to *B*-DNA. In the case of putrescine^2+^ the most probable positions are in (m14÷m18) and (m7÷m10) nucleotide regions of the minor groove. In the case of spermidine^3+^ molecule, the interaction sites are (m15÷m18) and (m6÷m10) in the minor groove and (j10÷j13) in the major groove. In the case of the system with spermine^4+^ molecules, there are two positions in the minor groove (m7÷m9) and (m14÷m16) where the localization of polyamine molecule is most probable. Taking into consideration the dependence of the minor groove width on the sequence of nucleotide bases (Figure 2), a clear correlation between polyamine positioning and minor groove width can be observed. Such correlation is better highlighted in Figure 4, where the width of the minor groove, averaged over all the simulated systems, is plotted and the most probable locations of the polyamines are marked. It can be seen that the polyamine molecules tend to be localized in the narrowest parts of the minor groove of the double helix in (m14÷m18) and (m6÷m10) nucleotide regions. These regions are centered around the two A-tracts.

Thus, the simulation results show that the polyamine molecules tend to be localized in DNA minor groove in the regions with the narrowest width. At the same time, the width of the minor groove of the double helix depends on the sequence of nucleotide bases. In the case of the simulated DNA sequence, the narrowest width of the minor groove is in the A-tract. Same results are observed both using CHARM27 and CHARMM36 force fields.

### Electrostatic model of polyamine interaction with the DNA double helix

**Figure 5.**
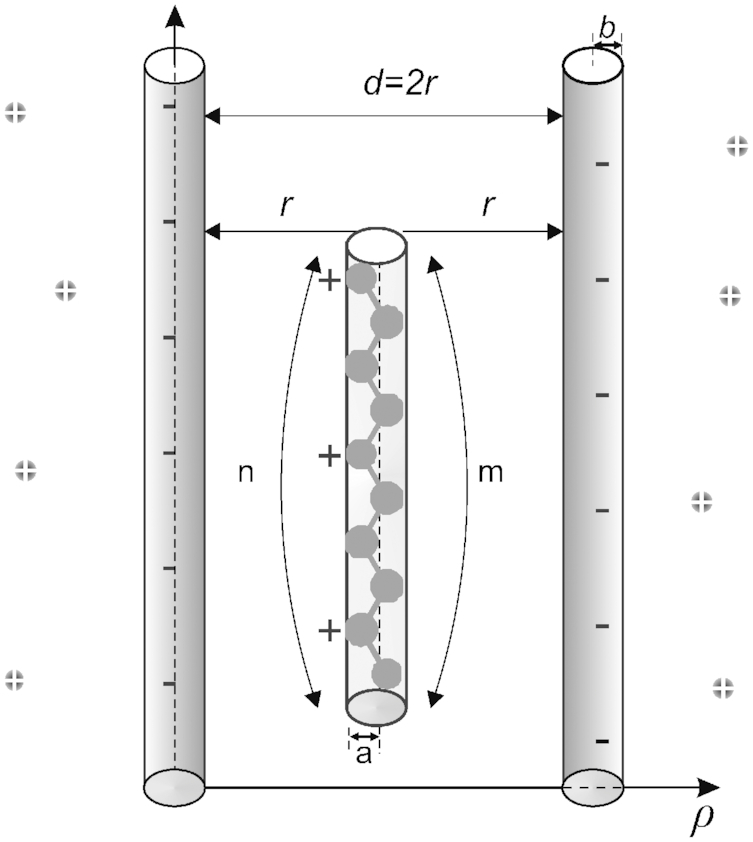
Model of polyamine interaction with DNA double helix. *r* is the distance from the center of the atomic group of the molecule to the charged chain; *m* is the number of atomic groups of polyamine molecule; *n* is the number of charged atomic groups in the molecule; *b* is the characteristic size of the phosphate group and the atomic group of the polyamine molecule. In the model spermidine^3+^ molecule is shown as an example.

To rationalize the sequence specificity of the DNA/polyamine interaction observed in the MD simulations, we developed a simple theoretical model. The model represents DNA as a pair of linear chains with negative charges corresponding to the phosphate backbone of the double helix (Figure 5). The space between the chains is constrained by the atomic groups of DNA (nucleotide bases, sugar rings, phosphate groups) and corresponds to the groove of the double helix. The width of the groove in this simple model is the distance between charged chains.

The free energy of such a system can be presented as a sum following contributions:

(2) \begin{equation*}{\rm{\Delta }}G = {\rm{\Delta }}{G_{el}} + {\rm{\Delta }}{G_{rep}} + {\rm{\Delta }}{G_0},\end{equation*}

where $\Delta {G_{el}}$ and $\Delta {G_{rep}}$ describe the electrostatic attraction and the repulsion, respectively, at small distances between DNA and polyamine molecule; $\Delta {G_0}$ contains the remaining contributions to the interaction free energy.

The electrostatic attraction appears due to the interaction of positively charged amino groups with the negatively charged phosphate groups of DNA backbone, and it may be described using different approaches, starting with simple polyelectrolyte models (37) to more detailed calculations within the framework of Poisson-Boltzmann equation (38). In the present work, we follow the concept of DNA ion-phosphate lattice which was developed for the description of the vibrations of DNA counterions (77–80). According to this approach, the backbone of DNA double helix is a lattice of charges consisting of the phosphate groups and counterions. To describe the electrostatic attraction and repulsion energy of DNA–polyamine interaction we use the Born-Mayer potential that is developed for the description of the energy of ionic crystals (81):

(3) \begin{equation*}U\left( \rho \right) = - \frac{{{M_a}{e^2}}}{{4\pi \varepsilon {\varepsilon _0}\rho }} + B{e^{ - \frac{\rho }{\kappa }}},\end{equation*}

where $e$ is the electron charge,${M_a}$ is the Madelung constant describing the interaction of the charge with the other charges of the system, $\varepsilon$ is the dielectric constant, $\rho$ is the coordinate describing the localization of the ion in the lattice point; $B$ and $\kappa$are the parameters describing the repulsion at small distances.

The potential energy presenting the electrostatic and repulsion interactions for the case of polyamine molecule between two charged chains (${\rm{\Delta }}{G_1} = {\rm{\Delta }}{G_{el}} + {\rm{\Delta }}{G_{rep}}$) in the Born-Mayer form (equation (3)) may be written as follows:

(4) \begin{eqnarray*}\Delta {G_1} = - n\frac{{{M_a}{e^2}}}{{4\pi \varepsilon {\varepsilon _0}}}\left( {\frac{1}{\rho } + \frac{1}{{2r - \rho }}} \right) + Bm\left( {{e^{ - \frac{\rho }{\kappa }}} + {e^{ - \frac{{2r - \rho }}{\kappa }}}} \right),\nonumber\\ \end{eqnarray*}

where $n$ is the number of charged amino groups in the polyamine molecule, $r$ is the distance from the center of the minor groove to the center of charged cylinder (DNA backbone, see Figure 5). In Eq. (4) the first term describes the electrostatic attraction of polyamine molecule with the first and the second phosphate chains of DNA double helix. The second term in Eq. (4) describes the repulsion at close distances between the atoms of polyamine molecules and phosphate groups and the exponents in brackets describe the repulsion from the first and the second strands of the DNA double helix, respectively. The interactions of charged amino groups between each other are considered the same in the minor groove of DNA and in the bulk; therefore, their contribution to the energy is neglected.

**Figure 6.**
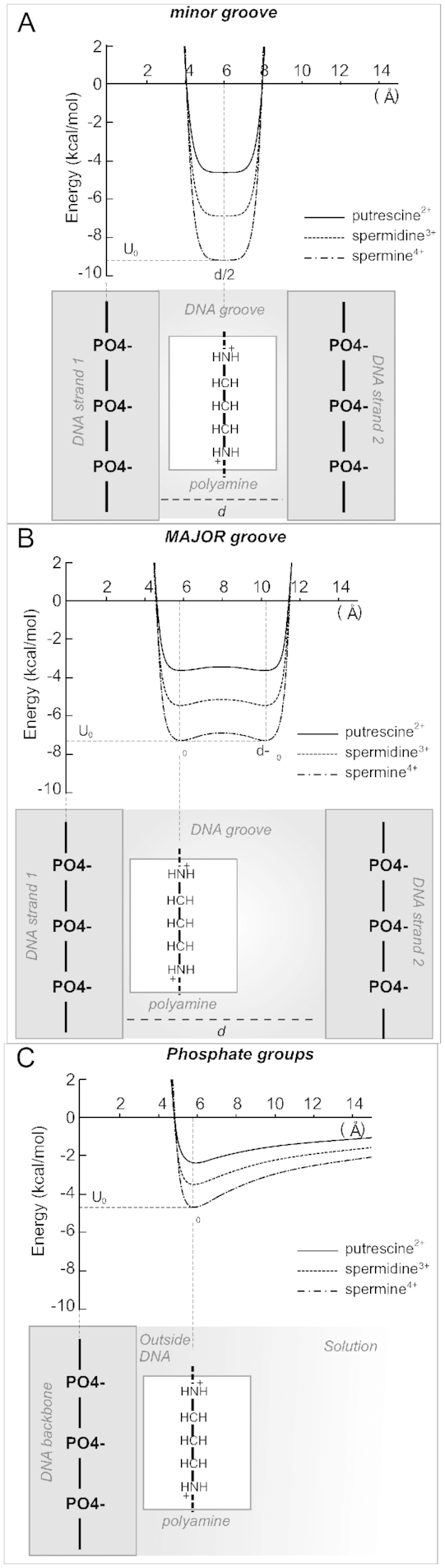
Dependence of the energy on the width of the grooves of the double helix for putrescine^2+^, spermidine^3+^ and spermine^4+^ molecules in complex with DNA. The cases of localization of polyamine molecule in minor groove (**A**), major groove (**B**), and near the phosphate group from the outside of the double helix (**C**). The figures at the bottom of each graph illustrate the scheme of localization of polyamine molecule with respect to the strands of the double helix (the size of the polyamine molecule and DNA backbone are shown proportionate to the width of the double helix groove).

The constant $B$ may be found from the equilibrium condition: ${ {\partial \Delta {G_1}/\partial \rho } |_{\rho = {\rho _0}}} = 0$, where ${\rho _0}$ is the equilibrium distance for the polyamine molecule and one of the negatively charged sugar-phosphate strands of the double helix:

(5) \begin{eqnarray*}B = \frac{n}{m} \cdot \frac{{{M_a}{e^2}}}{{4\pi \varepsilon {\varepsilon _0}}} \cdot \frac{{4r\left( {r - {\rho _0}} \right)\kappa }}{{\rho _0^2{{\left( {2r - {\rho _0}} \right)}^2}}} \cdot \frac{1}{{{e^{ - \frac{{{\rho _0}}}{\kappa }}} + {e^{ - \frac{{2r - {\rho _0}}}{\kappa }}}}}.\end{eqnarray*}

The third term (${\rm{\Delta }}{G_0}$) in Eq. (2) describes the contributions from hydration energy, conformational entropy, and mixing entropy. The variation in the hydration contribution occurs due to the substitution of water molecules of the DNA hydration shell by the polyamine molecule. The conformation energy is due to the change of the conformational state of the polyamine molecule. The contribution from the mixing entropy arises due to the increase of the local concentration of polyamine molecules near the DNA double. The hydration energy helps to stabilize the double helix due to the hydrophobic nucleotide bases (1,3), while both the conformational and mixing energies give destabilizing contributions by decreasing of the entropy inside DNA groove. In the present work we assume that the stabilizing and destabilizing terms in the energy ${\rm{\Delta }}{G_0}$ compensate each other, and in our further considerations it is set equal to zero.

Using the expression for the Bjerrum length ${L_B} = {e^2}/( {4\pi \varepsilon {\varepsilon _0}{k_B}T} )$ and taking into consideration the equations (4) and (5), the free energy of the system (equation (2)) normalized per ${k_B}T$ may be written in the form:

(6) \begin{eqnarray*}\Delta g &\approx& - n{M_a}{L_B}\left( {\frac{1}{\rho } + \frac{1}{{2r - \rho }}} \right)\nonumber\\ && + \frac{{4n{L_B}{M_a}r\left( {r - {\rho _0}} \right)\kappa }}{{\rho _0^2{{\left( {2r - {\rho _0}} \right)}^2}}} \cdot \frac{{{\rm{cosh}}\left( {\frac{{r - \rho }}{\kappa }} \right)}}{{{\rm{sinh}}\left( {\frac{{r - {\rho _0}}}{\kappa }} \right)}},\end{eqnarray*}

where ${k_B}$ is the Boltzmann constant; $\Delta g = \Delta G/{k_B}T$. In the equation (6) the first term describes the electrostatic attraction and the second term describes the repulsion between DNA and polyamine molecule.

To calculate the energy of polyamine molecule near the double helix by formula (6) the following parameters are needed: the parameter of repulsion ($\kappa$), the Madelung constant (${M_a}$) the equilibrium distance between polyamine molecule and backbone of DNA (${\rho _0}$) and the Bjerrum length (${L_B}$). The Madelung constant in the case of the dipole is 1, in the case of one-dimensional ionic lattice 1.386, and in the case of the NaCl ion lattice it is 1.748 (81). The values of ${M_a}$ estimated for the ion-phosphate lattice of DNA are about 1.1–1.5 depending on the counterion type (80). Varying the ionic strength may lead to a modest variation of the Madelung constant. The repulsion parameter $\kappa$ in the Born-Mayer potential is taken the same as in ionic crystals (81). The value of the Bjerrum length is calculated for the dielectric constant $\varepsilon = 78$ of bulk water and temperature 300 K (the temperature of simulated systems). The equilibrium distance is considered equal to the van der Waals radius of the phosphate group. All parameters used for the estimation of the free energy are shown in the Table 3.

**Table 3.** The parameters used in the calculation of the energy of polyamine molecule in the groove of the double helix

	Groove width ($d = 2r$), Å							
Polyamine	Minor groove	Major groove	*m*	*n*	${\rho _0}$	$\kappa$(80)	${L_B}$	${M_a}$ (80)
Putrescine^2+^			6	2				
Spermidine^3+^	12	17	10	3	5.8	3.28	7.7	1.5
Spermine^4+^			14	4				

The free energies estimated by equation (6) for the cases of localization of polyamine molecule in the narrow minor groove (assuming $d\, = \,12{\mathring{\rm A}}$) and wide major groove (assuming $d\, = \,17{\mathring{\rm A}}$) and outside of the double helix ($d = \infty$) are shown on the Figure 6. It is seen that the free energy functions may have different shapes. In the case of wide groove of the double helix (major groove) two potential wells are formed describing the interactions with the first and the second strands of the double helix. Thus, in the case of a wide groove (the major groove of *B*-DNA) the polyamine molecule may be localized near one of the strands of the phosphate backbone (Figure 6B). It is also possible to form the cross-strand links by polyamine molecule. In the case of a decreased width of the groove (minor groove of *B*-DNA) there is one single-well potential that is deeper compared to the major groove. Thus, the polyamine molecule in the minor groove is constrained by the phosphate backbone and form more stable complex with DNA (Figure 6A). In the case of localization of the polyamine molecule near the phosphate backbone from the outside of the double helix the free energy function has one small potential well that gradually diminishes as the distance increases (Figure 6C). The values of the free energies in the minimum of the potential wells are shown in the Table 4.

**Table 4.** The values of free energy of polyamine molecules in different regions of the double helix calculated using equation (6) (in kcal/mol). In the round brackets the values obtained from molecular dynamics simulations (34) are shown. The range of experimental energy values (30) is indicated in square brackets

Polyamine	Minor groove	Major groove	Phosphate group	Experiment (30)
Putrescine^2+^	–4.6	–3.5 (–7.1±3.7)	–2.3	–[5.781÷6.808]
Spermidine^3+^	–6.9 (–11.1±6.4)	–5.3	–3.5	–[5.821÷7.602]
Spermine^4+^	–9.2	–7.0	–4.7 (–13.7±6.5)	–[6.60÷8.441]

It is seen that in the case of the minor groove the polyamine molecules have ∼25% lower energy than that in the major groove and about twice lower than that outside the double helix. The obtained free energies of polyamine localization in the grooves of the double helix show that the ability of polyamine to interact with DNA has the following preference: spermine^4+^ > spermidine^3+^ > putrescine^2+^. This result agrees with our molecular dynamics simulation data where the same hierarchy of the residence times of polyamine molecules in the regions of the double helix (equation (1)) was observed (Table 1). The absolute values of interaction energies show reasonably good agreement with the calorimetric data (30). In the introduced model stabilizing and destabilizing contributions in $\Delta {G_0}$ compensate each other and the behavior of the free energy is governed by the electrostatic and repulsion contributions. The comparison of our results with energies obtained from molecular dynamics simulations (22) shows an agreement within the estimated errors.

**Figure 7.**
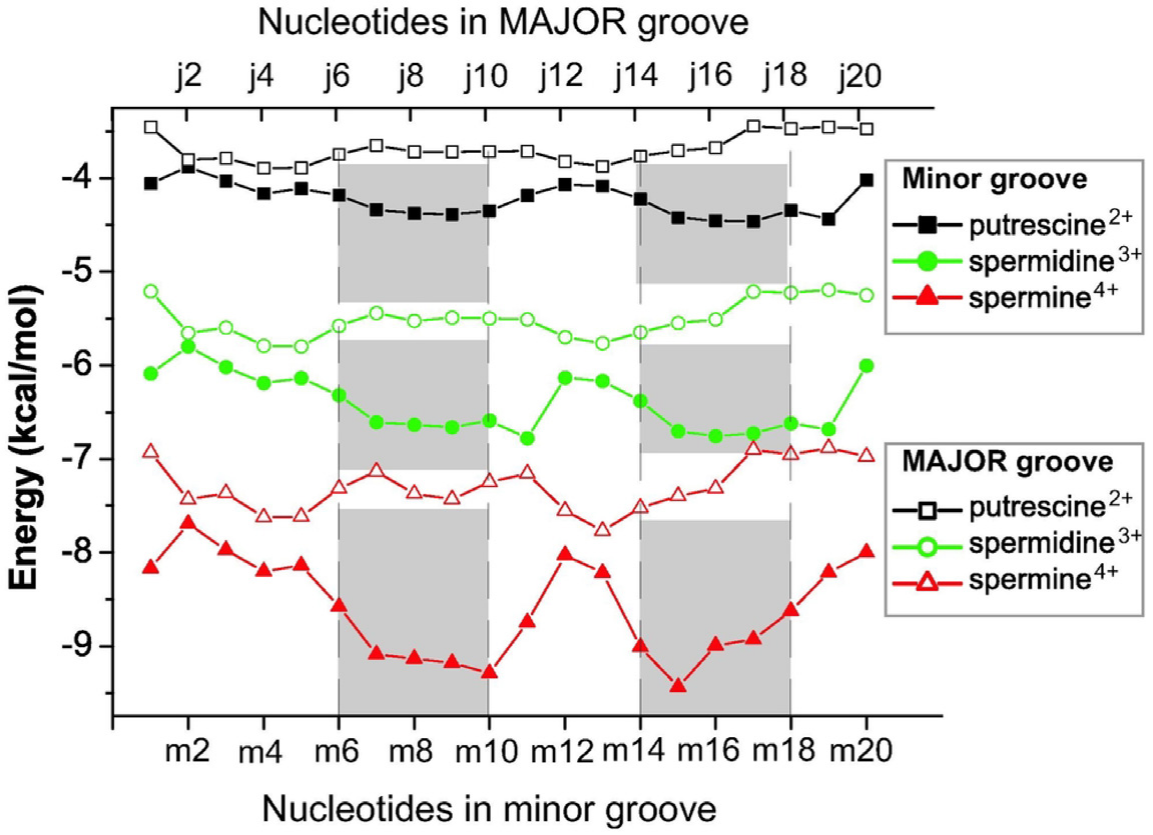
Dependence of the free energy on the width of the minor and major grooves of the simulated DNA fragment. The energies were calculated using equation (6) for the values of the widths of the grooves represented in Figure 2. The numeration of the nucleotide steps from the minor and major groove sides are shown according to Figure 1B. The places of the most probable localization of the polyamine molecules determined from molecular dynamics simulations are shown by gray bands.

Using the developed model, the interaction free energy of polyamines in the minor and major grooves was calculated as a function of the groove widths that were calculated from the MD simulations. The results point out a prominent correlation between the energy values and the width of the grooves (Figure 7). The free energy has its lowest values in the narrow minor groove and the highest in the wide major groove. The comparison of the results obtained using the developed theoretical model with those obtained from MD simulations shows that the nucleotide sequences, corresponding to the lowest free energy calculated by using the formula (6) are the most probable interaction sites for all of the three studied polyamines.

Furthermore, the developed model shows that the affinity of polyamines to the DNA macromolecule increases with the charge of the polyamines. The localization of polyamines in the narrow groove is energetically more favorable than in the wide groove and at the periphery of the double helix. Therefore, the polyamine molecules tend to be localized in the minor groove of *B*-DNA double helix. The developed theoretical model provides a qualitative description of DNA–polyamine interaction.

## DISCUSSION

**Figure 8.**
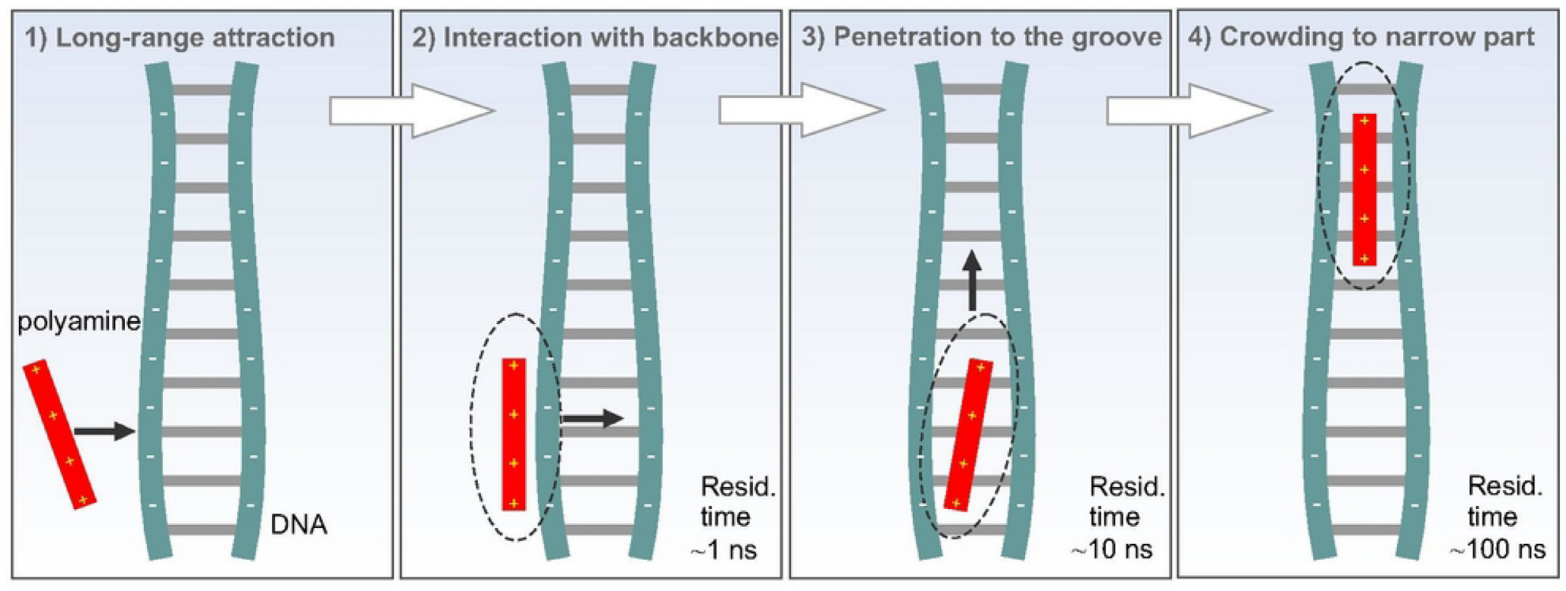
Scheme of polyamine interaction with DNA double helix.

Since the phosphates of the double helix are the groups most exposed to the solution, the interaction of polyamines with DNA starts preferably by contacts with these groups. After the formation of polyamine–phosphate complexes the polycationic molecules move into one of the grooves of the double helix or return back to the solution. The polyamines prefer to populate the narrowest part of the grooves that is governed by the minimum of electrostatic energy. The widths of the minor and major grooves of DNA double helix are not constant but show thermal fluctuations and a dependence on the nucleotide sequence (1). The amplitude of the thermal fluctuations of the groove width is ∼1Å (Figures 2B and Supplementary Figure S2B) that is about the same as the amplitude of DNA conformational vibrations, observed in the low-frequency spectra (77–80). The change of the groove width with the nucleotide sequence is ∼2 Å or higher, which means that the width of DNA grooves is modulated by the nucleotide sequence. The lowest width is observed in minor groove of A-tracts, while the largest in the major groove of at the CG nucleotide sequence (Figures 2 and Supplementary Figure S12). Such sequence dependence of the groove width is well known, and in particular is known that A-tract have a much narrower minor groove than canonical *B*-DNA, and that this narrow groove contains an ordered network of water molecules called the spine of hydration (1,3,67). The presence of a 5′-TpA-3′ in AT rich sequence leads to a different hydration, and to the enlargement of the minor groove. Visual examination indicates that due to this sequence dependent effect, the polyamine molecules crawl in the groove of the double helix to find the narrow space it occupies with longer residence times. The scheme of the complex formation of polyamine molecule with the DNA groove is depicted on the Figure 8.

It is interesting to note that the narrowing of the groove at A-tracts is observed also at physiological ionic strength, and that in the ionic strength ranging from zero added salt to physiological, the value of Madelung constant is not largely affected by the ions concentration. Therefore, our interaction energies should be valid also in physiological condition. The distribution of polyamines and monovalent ions around the double helix is interdependent due to the long-range electrostatic interactions. In a recent study (36) it was found that increasing the concentration of NaCl leads to a reduction of the Sm^4+^ concentration, and this in turn could lead to a reduction of the polyamine binding to the minor groove; however, further simulations will be required to check this effect thoroughly.

Previous calorimetric studies for the systems of DNA with putrescine^2+^, spermidine^3+^ and spermine^4+^, respectively, indicate that the interaction of polyamine molecules with AT rich motifs of DNA is preferred (25,30). The suggested reason of such behavior was the different organization of the hydration shell of the double helix for different nucleotide sequence (30). According to our model the electrostatic contribution alone can already explain this behavior; however, considering the assumption made in our model, some contributions due to different hydration patterns cannot be excluded. This result is also in agreement with the experimentally observed magnification of the affinity of polyamine to DNA as the charge of polyamine molecule increases (30).

The polyamine preferential binding for the minor groove revealed by the present study was not revealed by previous MD simulations (36,53–56,64,65). The reason for this may in some cases be due to the short simulation times or in the simulated base pair sequences that were used in those simulations which either did not contain A-tract, or had too long sequence of A-tract. In works (53–56), the simulation trajectories were not longer than 5 ns; based on the presented results, such short lengths most likely do not allow to collect the necessary statistics of DNA–polyamine interactions. In work (64) the DNA fragment with CG motif of nucleotides was studied. A study of interaction of spermine^4+^ with *B*-DNA and RNA in *A*-form of the double helix was performed with respect to the condensation induced by polyamine molecules (65). In this study the atoms of *B*-DNA were constrained during whole simulation trajectory, and this removes the natural fluctuations of the groove widths keeping the grooves uniform along the double helix. In the same study, the atoms of RNA molecule and polyamines were free to move, and as a result, polyamines were found in the major groove of the double helix which, indeed, is the narrowest groove in the case of A-form of the double helix (1). In the work (66), *B*-DNA fragment poly(dA)poly(dT) with spermine^4+^ molecules was modeled and the localization of polyamines in the minor groove was found as the most preferable. To conclude, our analysis of previous simulation works shows that in works (53–56,65) the effect of polyamine being preferably localized in the A-tract of the minor groove was not observed due to chosen specific features in the simulation, while in the works (36,66) the certain sequence specificity of polyamine localization on DNA double helix could be detected.

Our simulations indicate a significant preferential pattern of binding of polyamines in the A-tract of DNA minor groove. This finding is expected to be important for the understanding of biological mechanisms of DNA functioning in living cells. The A-tract is known as the characteristic sequence pattern in the gene to which the proteins (histones and other DNA binding proteins) typically bind (82–86). The indirect recognition of A-tract by proteins occurs due to the characteristic structural features of the double helix and due to the ordering of water molecules in hydration hell (85,86). The hydration shell in the minor groove in A-tract region is known to be characterized by the spine of water molecules bridging the atoms of nucleotide bases of different strands (83,85–88). The localization of highly charged polyamine molecule in the minor groove of the double helix disturbs the structure of the hydration spine making the minor groove in this region not as electronegative as in the case of the absence of the polyamine molecule in it. The bendability of DNA in AT region should be affected by polyamines distinguishing this sequence motif from others and inducing some modulation of the macromolecule elasticity with respect to nucleotide sequence. Thus the preferential localization of polyamine in the A-tract of DNA minor groove should have an impact on the mechanisms of nucleic-protein recognition and DNA compaction in the chromatin.

While the concentration of polyamines used in our simulation is about one order higher than that reported for living cells, the possibility of abundant local concentration of polyamines for AT-rich regions, as well as deficiency of polyamines in AT-depleted regions warrants further consideration. In this case the atomistic simulations should be extended by coarse grained modeling due to a significant rise of the computational cost and the both computational and experimental methods at the mesoscopic scale should be used. At the same time, to describe the details of interaction of polyamines in the binding sites of DNA and proteins and possible manifestations in the experiments, calculations of the electron structure can give important insights. With this respect, for the further studies of the mechanisms of impact of polyamines in nucleic-protein interactions and the compaction of DNA in chromatin a multiscale approach should be used from atomistic to macromolecular level.

## CONCLUSIONS

MD simulations of a series of polyamine/DNA systems in water solution were carried out to investigate the pattern preferences of *B*-DNA nucleotide motifs by putrescine^2+^, spermidine^3+^ and spermine^4+^ molecules. The interaction of polyamine with DNA starts by coming into contact with the sugar-phosphate backbone, and from there the polycationic molecule can move to one of the grooves (minor grooves or major groove). The hierarchy of the residence times of polyamine molecules in different DNA binding sites was established: the longest residence time (∼100 ns) is observed in the minor groove, while in the major groove the polyamines reside shorter time (∼10 ns), and in the case of the interaction of polyamines with the phosphate groups from the outside of the double helix, the residence time is the shortest (about 1 ns). Same trend is common to all the considered polyamines. By comparing the polyamines with each other, the stability of DNA–polyamine complexes increases as the charge of polycations increases. The polyamine molecules prefer to be localized in the groove of the double helix characterized by the lowest width. The widths of grooves of the double helix were found to depend on the sequence of nucleotide bases, and this in turn induces the polyamine to bind preferentially to some nucleotide sequence motifs. In the case of the considered DNA fragment the narrowest region is the minor groove in A-tract, and the polyamines were found to reside the longest time in this region. To further characterize the binding preference, we developed a simple theoretical model, which allowed estimating the free energy of the system as a function of the polyamine localization. The estimated energies of the system show that the narrowest groove of the double helix is energetically favored in the interaction with the polyamines and that the electrostatics is the dominant contribution to DNA–polyamine interaction. The obtained results agree with existing experimental data and previous MD simulations. Thus, in the presented study the preferable localization of putrescine^2+^, spermidine^3+^ and spermine^4+^ in the minor groove with A-tracts is discussed and found to be due to the modulation of the groove width by the nucleotide sequence.

## Supplementary Material

gkz434_Supplemental_Files
