# Supplementary material for: Pattern preferences of DNA nucleotide motifs by polyamines putrescine2+, spermidine3+ and spermine4+
Source: Nucleic Acids Res. 2019 May 22;47(12):6084–97. doi: 10.1093/nar/gkz434 (PMC6614828; doi:10.1093/nar/gkz434)
Supplement: gkz434_Supplemental_Files [file gkz434_supplemental_files.pdf]

Supplemental materials

Pattern preferences of DNA nucleotide motifs by polyamines putrescine^{2+}, spermidine^{3+}, and spermine^{4+}

Sergiy Perepelytsya[1,2,*,†], Jozef Uličný[3, †], Aatto Laaksonen[4,6] and Francesca Mocci[5,6]

[1]Bogolyubov Institute for Theoretical Physics of the National Academy of Sciences of Ukraine, 03143 Kyiv, Ukraine
[2]Department of Theoretical and Mathematical Physics, Kyiv Academic University, 03142 Kyiv, Ukraine
[3]Department of Biophysics, Institute of Physics, P. J. Šafárik University, 041 54 Košice, Slovakia
[4]Division of Physical Chemistry, Department of Materials and Environmental Chemistry, Arrhenius Laboratory, Stockholm University, 10691 Stockholm, Sweden
[5]Department of Chemical and Geological Sciences, University of Cagliari, I-09042 Monserrato, Italy
[6]Centre of Advanced Research in Bionanoconjugates and Biopolymers, Petru Poni Institute of Macromolecular Chemistry, Iasi, 700487, Romania

* To whom correspondence should be addressed. Tel: +38-044-521-3495; Fax: +38-044-526-5998; Email: perepelytsya@bitp.kiev.ua

[†]These authors contributed equally to this work as first authors.

Section S1. System parameters and simulation protocol

The simulation was started with a minimization of the system with fixed heavy atoms. Then the system was minimized with fixed DNA, polyamine atoms, and ions. After minimization water component of the solvent was heated to the temperature 300 K and equilibrated. Then the system with fixed DNA and polyamine atoms, and constrained ions was heated to the temperature 300 K and then equilibrated (the first with restrained and then with free ions). After that, the system was minimized with fixed DNA atoms and restrained polyamine atoms, then it was heated to the temperature 300 K and equilibrated (the first with restrained and then with free atoms). Then the atoms of DNA were allowed to move with some restrains and the minimization with following heating to the temperature 300 K was done. Then the system was equilibrated (the first with restrained and then with free DNA atoms). The simulations at these stages were performed at the constant pressure (101.325 kPa). At the production stage the systems were simulated at constant volume and temperature (NVT ensemble) during 1000 ns.

Table S1. Parameters of the simulated systems of DNA with polyamines.

System name	Polyamine molecule	Number of polyamine molecules	Number of water molecules	Number of counterions (Na^+)	Box size (L_x L_y L_z)
Put-DNA	Put^{2+}	4	5485	32	47 56 72
Spd-DNA	Spd^{3+}	3	5481	31	52 51 72
Spm-DNA	Spm^{4+}	2	5487	32	53 49 71

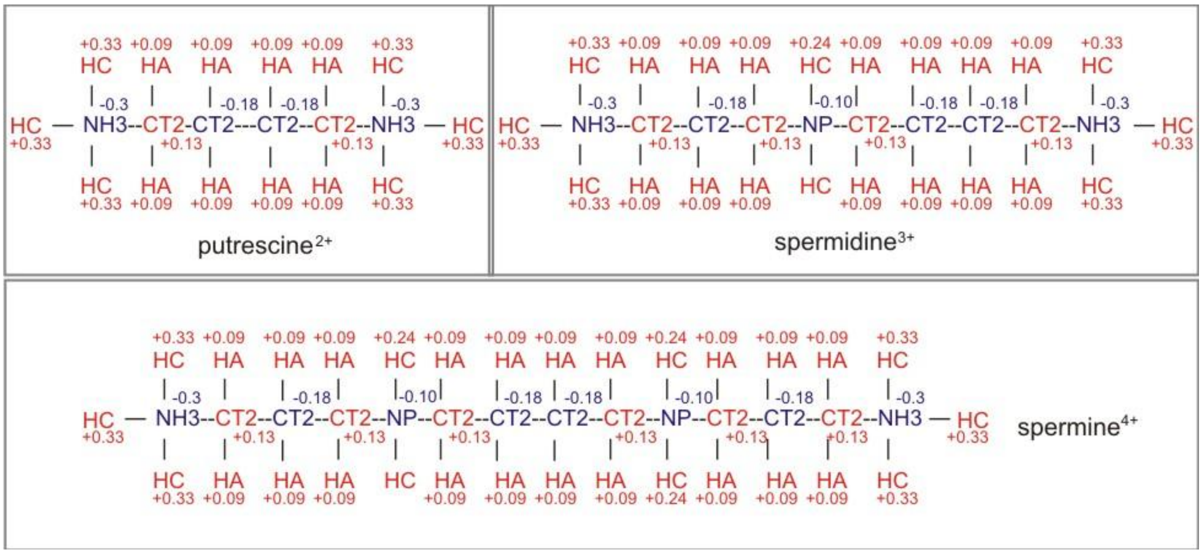

Figure S1. The atoms of polyamine molecules according to the scheme of CHARMM22 force field. The charge values are shown near the atoms. The atoms with positive and negative partial charges are colored in red and blue, respectively.

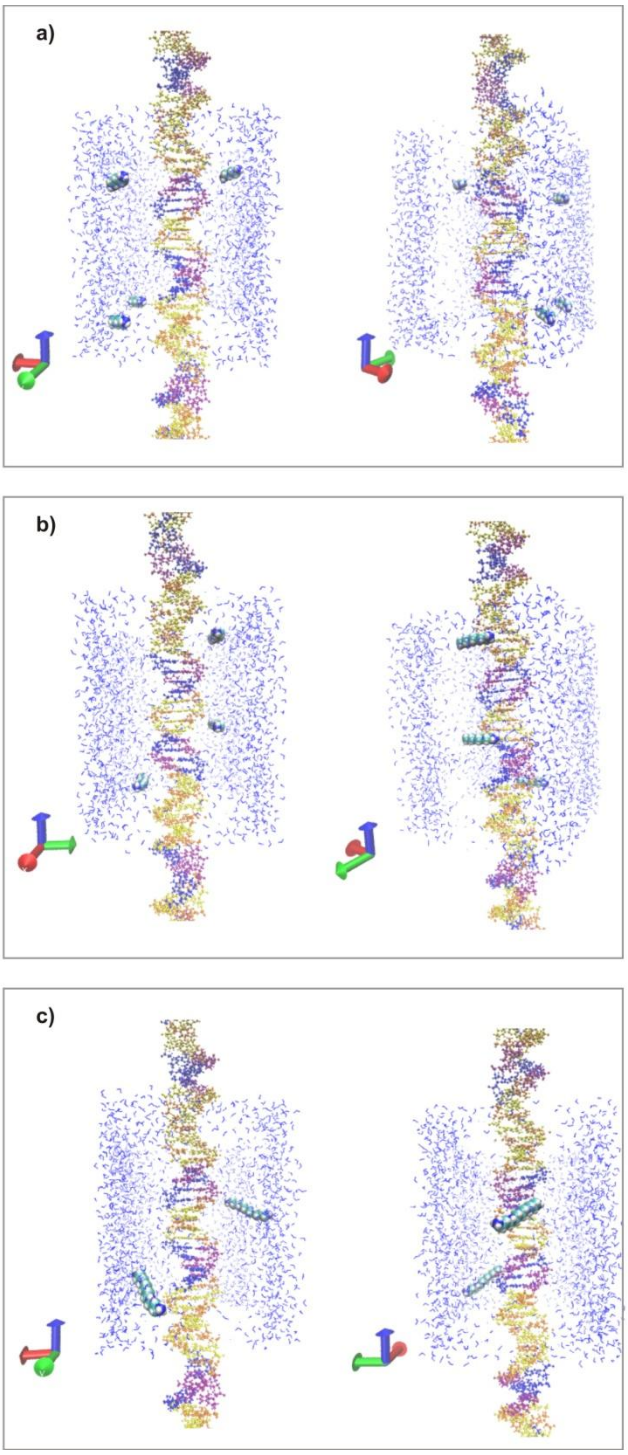

Figure S2. Initial systems in different representations. a) DNA with putrescine^{2+}. b) DNA with spermidine^{4+}. c) DNA with spermine^{4+}.

Table S2. Protocol of simulation of DNA-polyamine systems. k is the restrain coefficient (kcal/molÅ^2); r is temperature increment for equilibration (ºK/step); T is the temperature (ºK).

Step	Procedure	DNA	Polyamine	Water	ions	Step (fs)	Number of steps
1	Minimization	Fixed	Fixed	Fixed O	Fixed	-	2000
2	Minimization	Fixed	Fixed	Free	Fixed	-	2000
3	Heating	Fixed	Fixed	Free	Fixed	1	15000 (r=0.02)
4	Equilibration: NPT ensemble	Fixed	Fixed	Free	Fixed	1	100000
5	Heating: T=300	Fixed	Fixed	Free	Constrained (k=5)	1	15000 (r=0.02)
6	Equilibration: NPT ensemble	Fixed	Fixed	Free	Constrained (k=5)	1	100000
7	Equilibration	Fixed	Fixed	Free	Free	1	100000
8	Minimization	Fixed	Free	Free	Free	-	2000
9	Heating: T=300	Fixed	Constrained (k=5)	Free	Free	1	30000 (r=0.02)
10	Equilibration: NPT ensemble	Fixed	Constrained (k=5)	Free	Free	1	100000
11	Equilibration: NPT ensemble	Fixed	Free	Free	Free	1	100000
12	Minimization	Free	Free	Free	Free	-	2000
13	Heating: T=300	Constrained (k=5)	Free	Free	Free	1	30000 (r=0.02)
14	Equilibration: NPT ensemble	Constrained (k=5)	Free	Free	Free	1	500000
15	Equilibration: NPT ensemble	Free	Free	Free	Free	1	500000
16	Equilibration: NVT ensemble	Free	Free	Free	Free	2	$5*10^8$

Section S2. Simulation results for DNA with purtrescine^{2+}, spermidine^{3+}, and spermine^{4+} with use of CHARMM27 force field

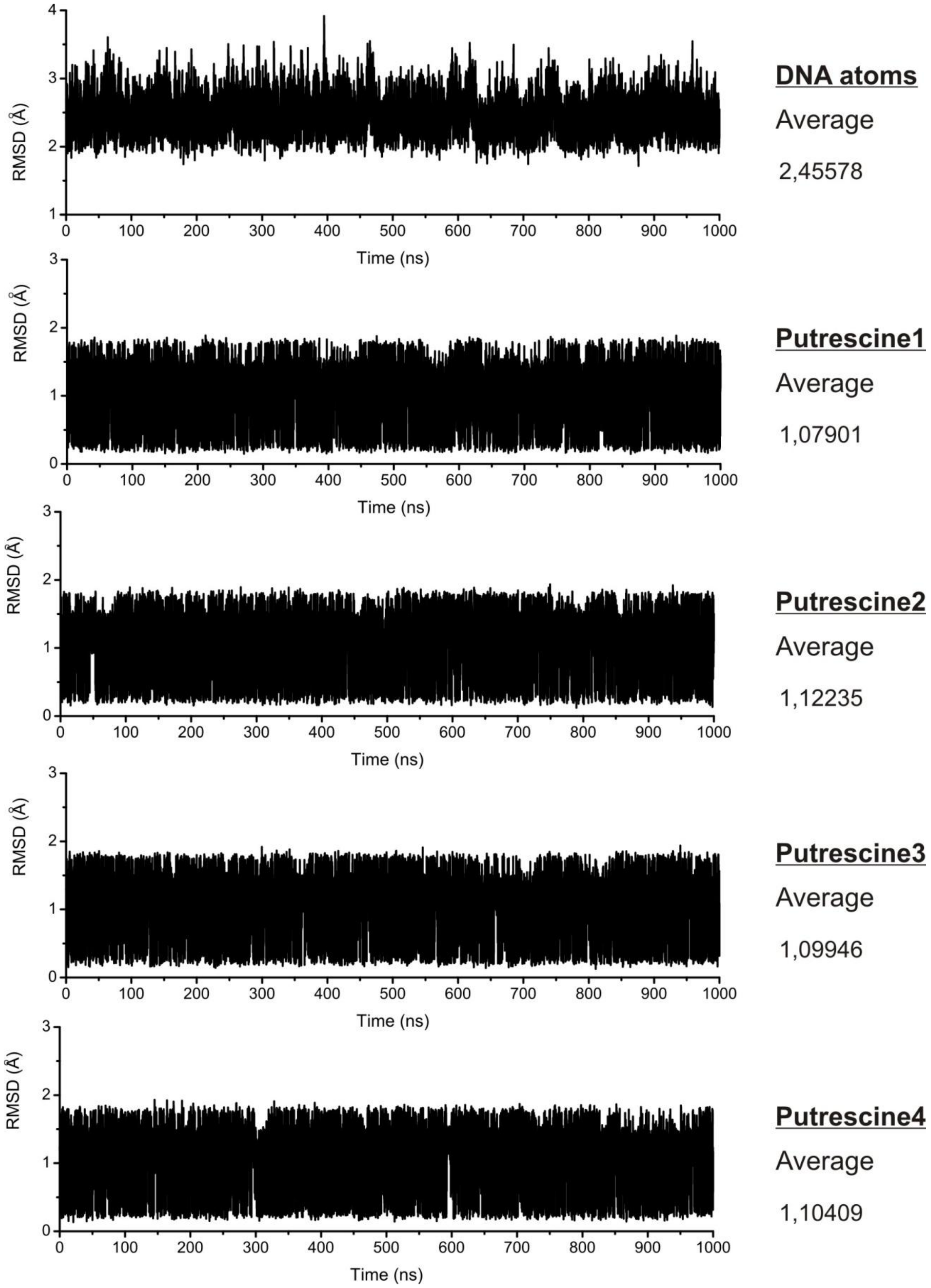

Figure S3. The root mean square deviations (RMSD) of DNA with putrescine^{2+} molecules. CHARMM27 force field.

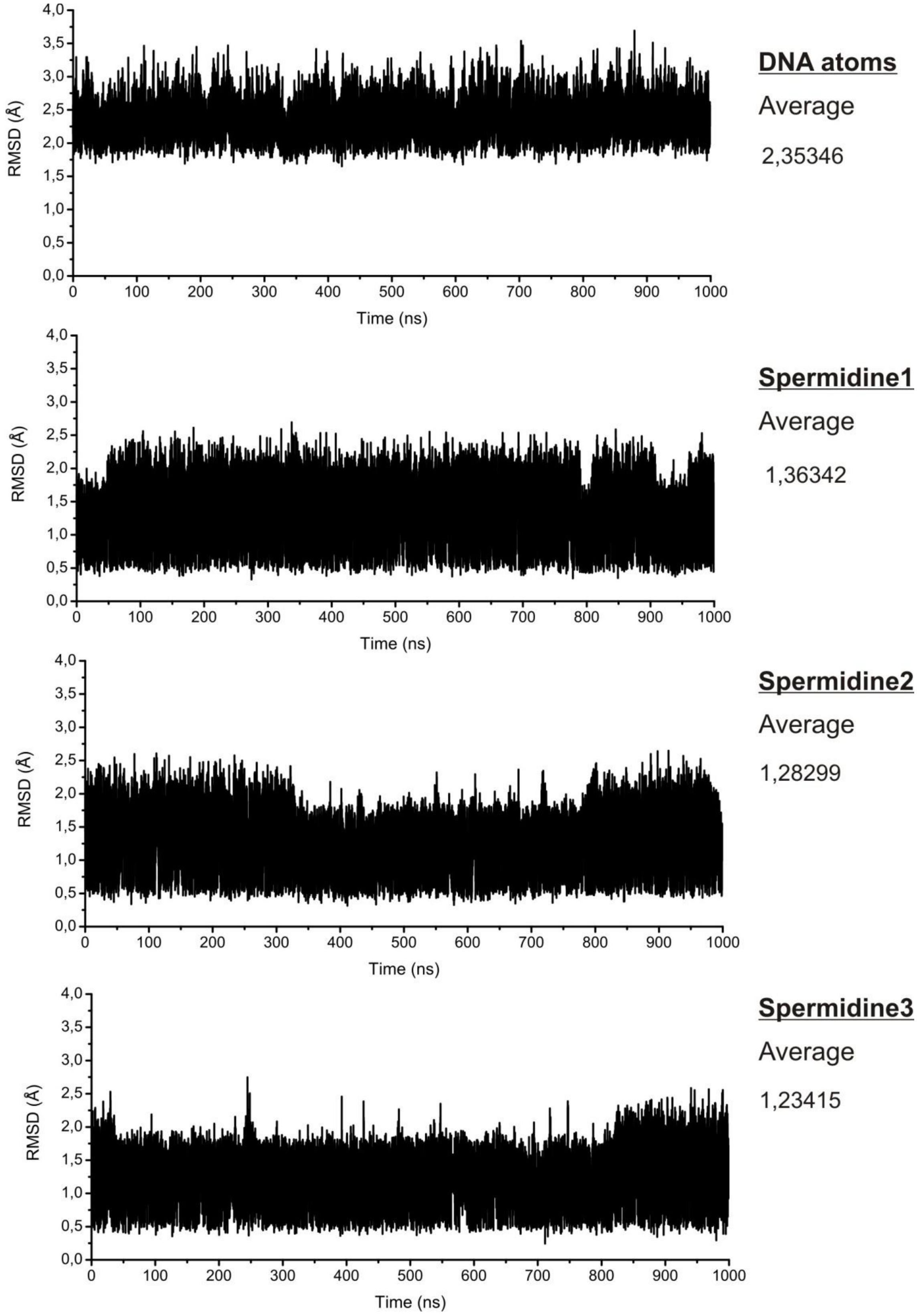

Figure S4. The root mean square deviations (RMSD) of DNA with spermidine^{3+} molecules. CHARMM27 force field.

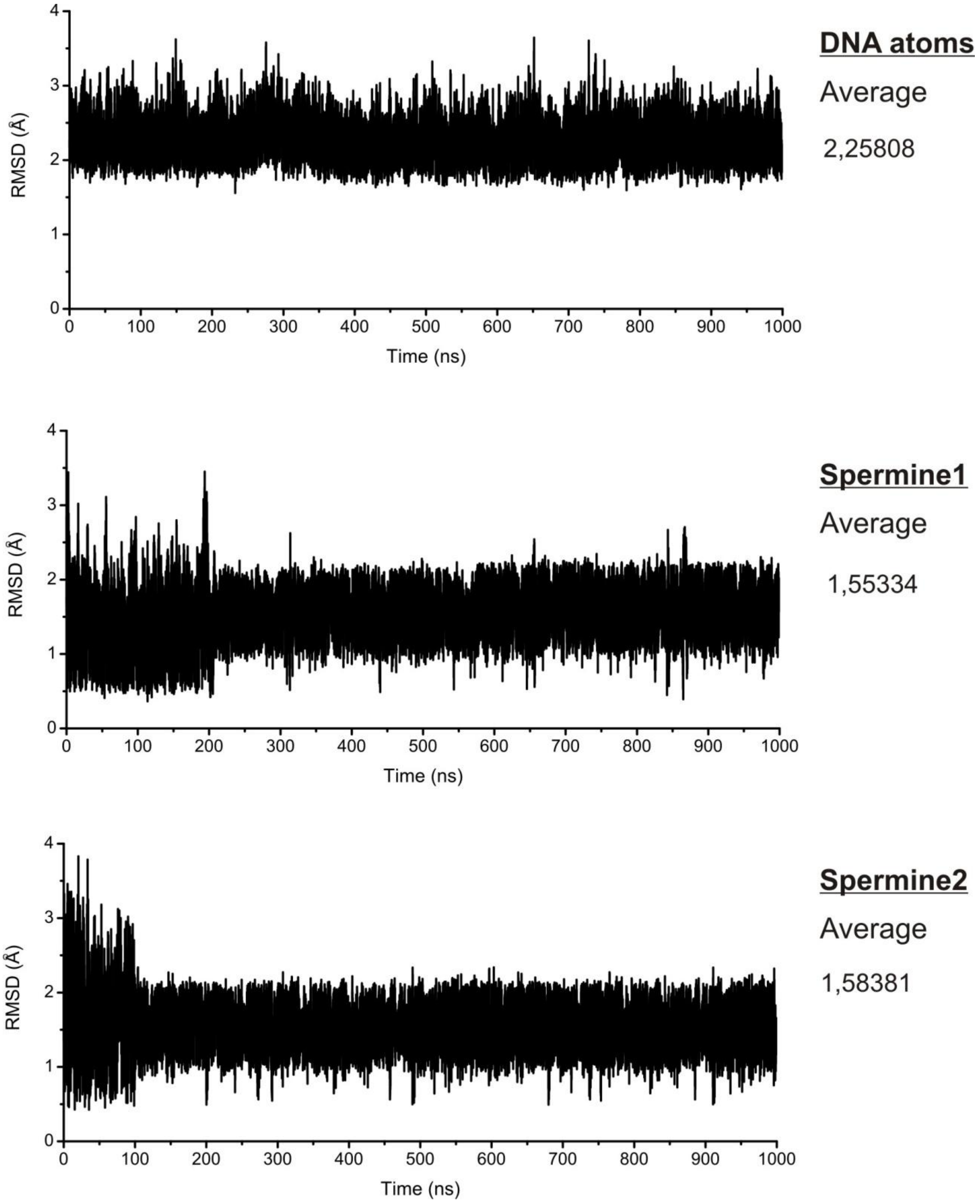

Figure S5. The root mean square deviations (RMSD) of DNA with spermine^{4+} molecules. CHARMM27 force field.

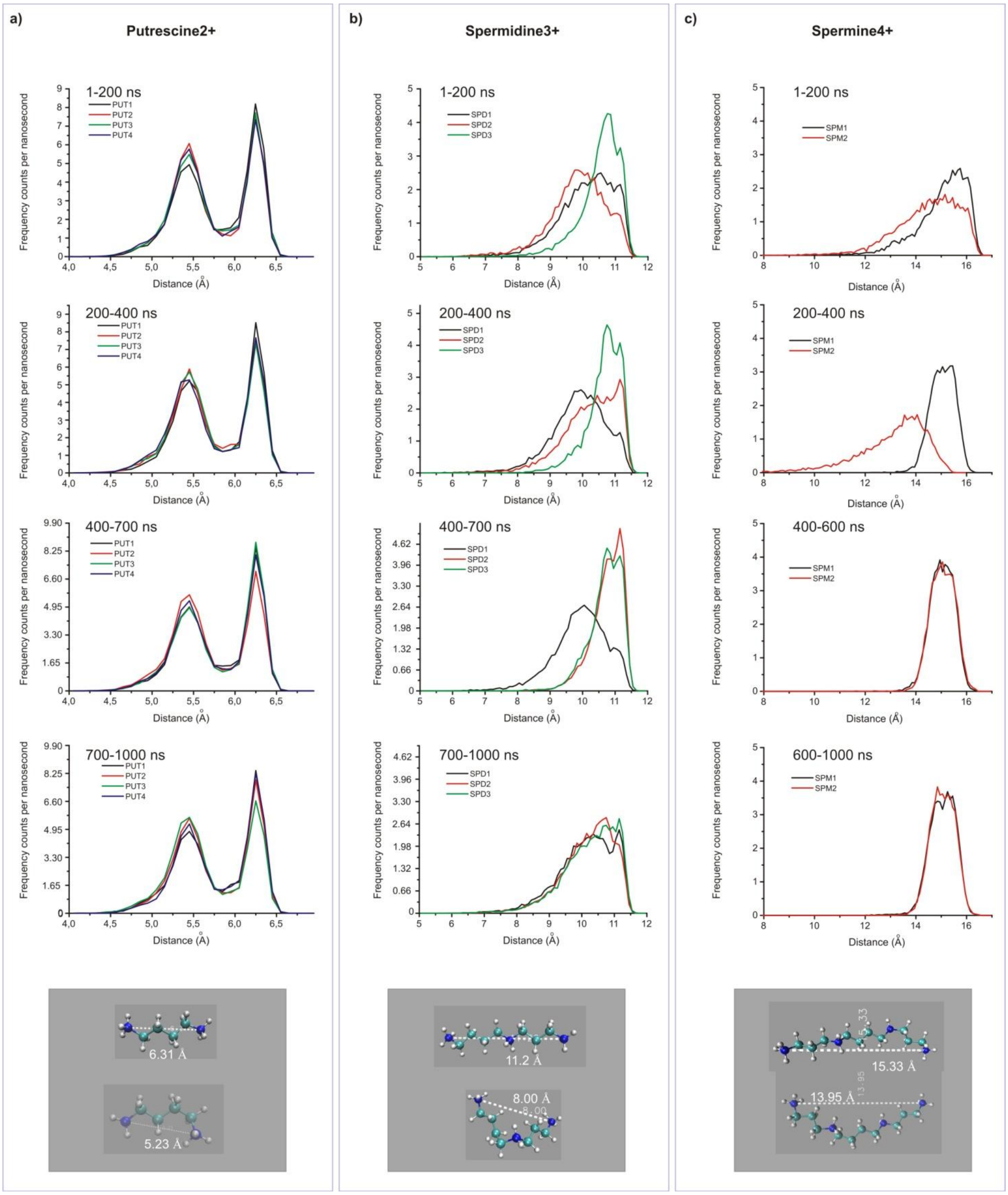

Figure S6. The distributions of the distances between the ends of polyamine molecules in the case of putrescine (a), spermidine (b), and spermine (c). The curves of the distribution correspond to each polyamine molecule in the system. At the bottom the snapshots of the molecules in characteristic states are shown. CHARMM27 force field.

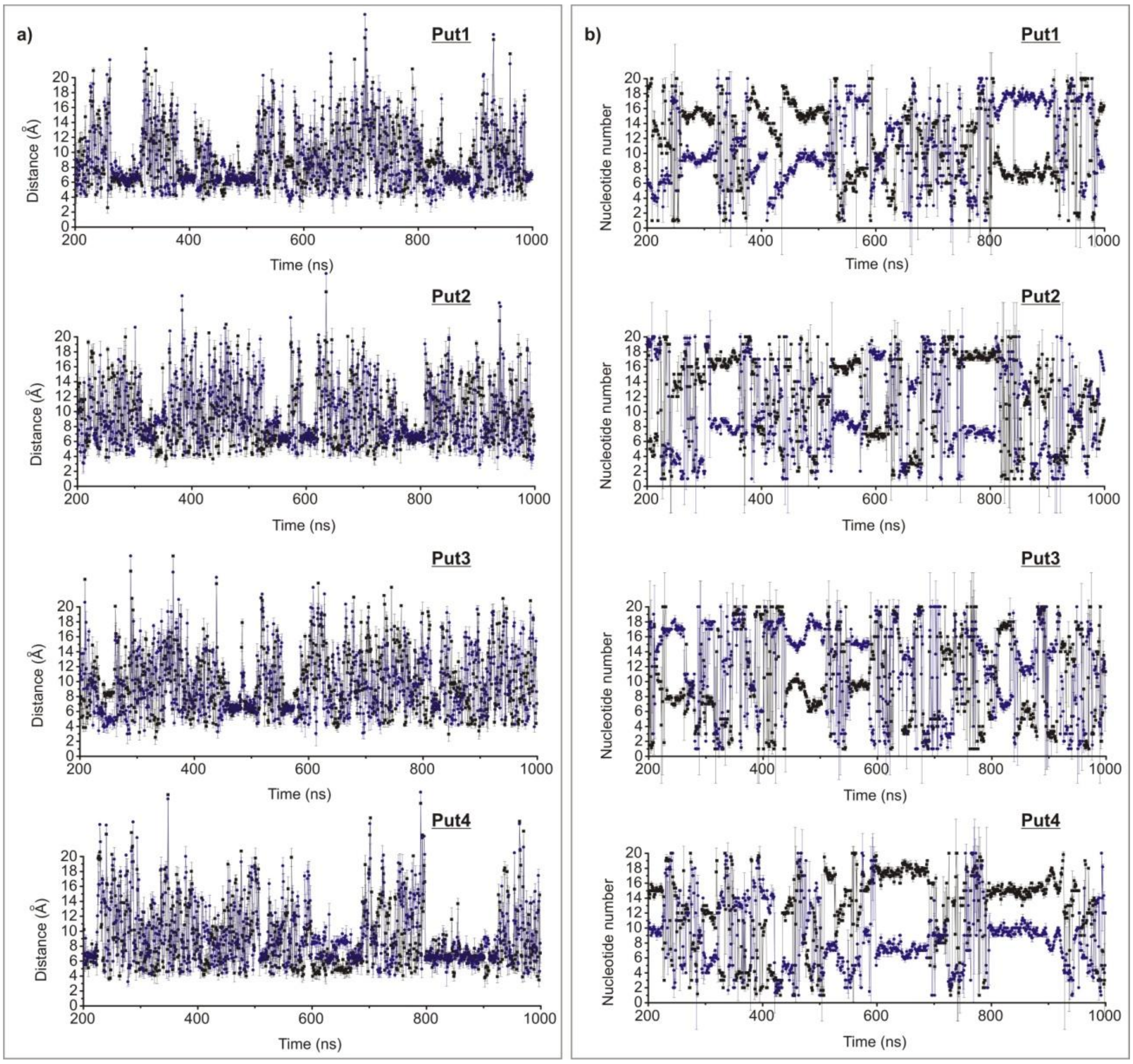

Figure S7. Localization of polyamine molecules with respect to the phosphate groups of the double helix in PutDNA system. a) The time dependence of average distance from polyamine molecule to the phosphate group of the double helix (black line – chain1; blue line – chain2). The distance was averaged over all atoms of the polyamine backbone at each nanosecond of simulation tragectory. b) The time dependence of the phosphate group number which is nearest to the polyamine molecule (black line – chain1; blue line – chain2). The value of the phosphate group number was averaged over all atoms of the polyamine backbone at each nanosecond of simulation tragectory. CHARMM27 force field.

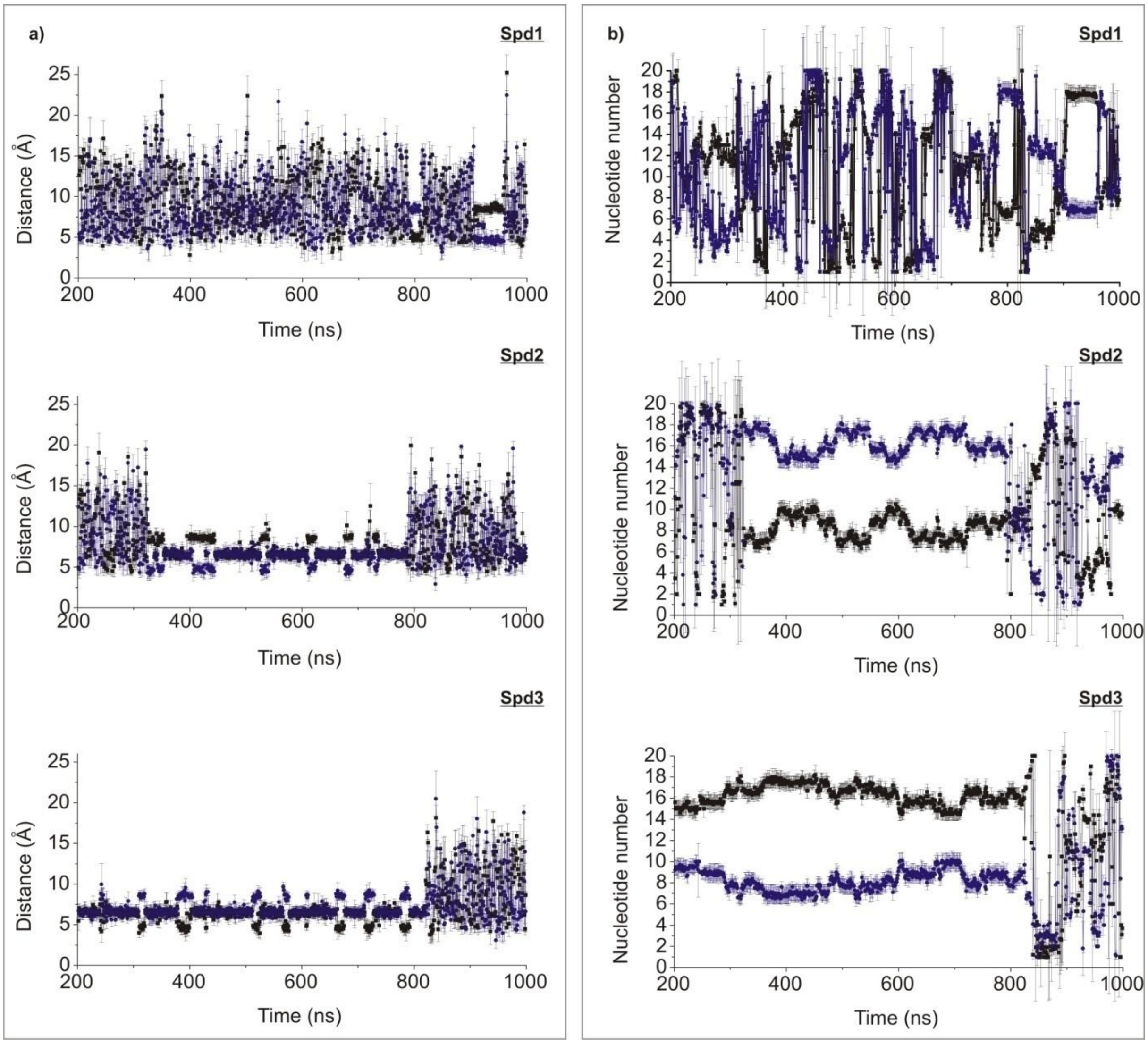

Figure S8. Localization of polyamine molecules with respect to the phosphate groups of the double helix in SpdDNA system. a) The time dependence of average distance from polyamine molecule to the phosphate group of the double helix (black line – chain1; blue line – chain2). The distance was averaged over all atoms of the polyamine backbone at each nanosecond of simulation tragectory. b) The time dependence of the phosphate group number which is nearest to the polyamine molecule (black line – chain1; blue line – chain2). The value of the phosphate group number was averaged over all atoms of the polyamine backbone at each nanosecond of simulation tragectory. CHARMM27 force field.

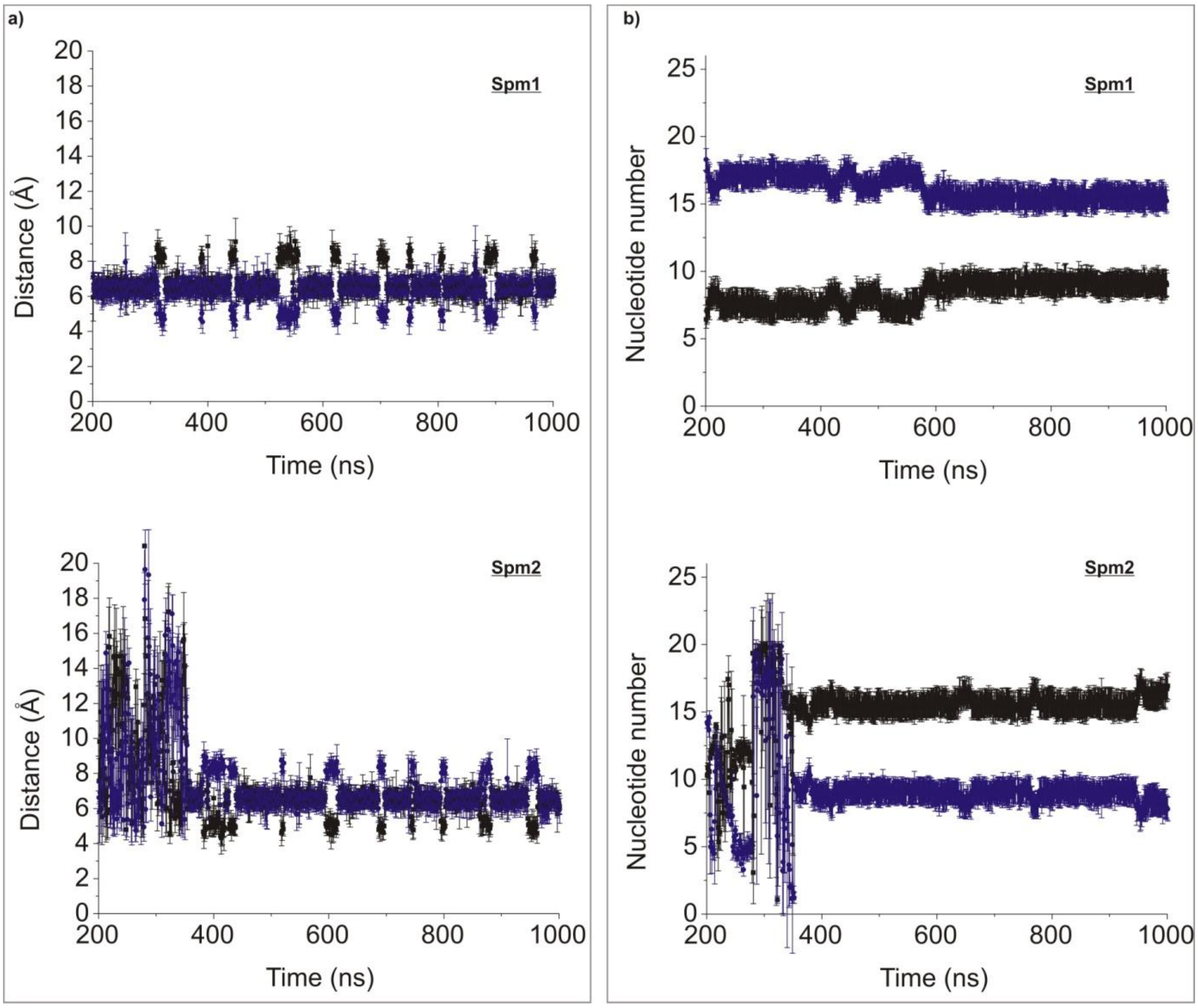

Figure S9. Localization of polyamine molecules with respect to the phosphate groups of the double helix in SpdDNA system. a) The time dependence of average distance from polyamine molecule to the phosphate group of the double helix (black line – chain1; blue line – chain2). The distance was averaged over all atoms of the polyamine backbone at each nanosecond of simulation tragectory. b) The time dependence of the phosphate group number which is nearest to the polyamine molecule (black line – chain1; blue line – chain2). The value of the phosphate group number was averaged over all atoms of the polyamine backbone at each nanosecond of simulation tragectory. CHARMM27 force field.

Section S3. The results of simulations for DNA with spermidine^{3+} with use CHARMM36 force field

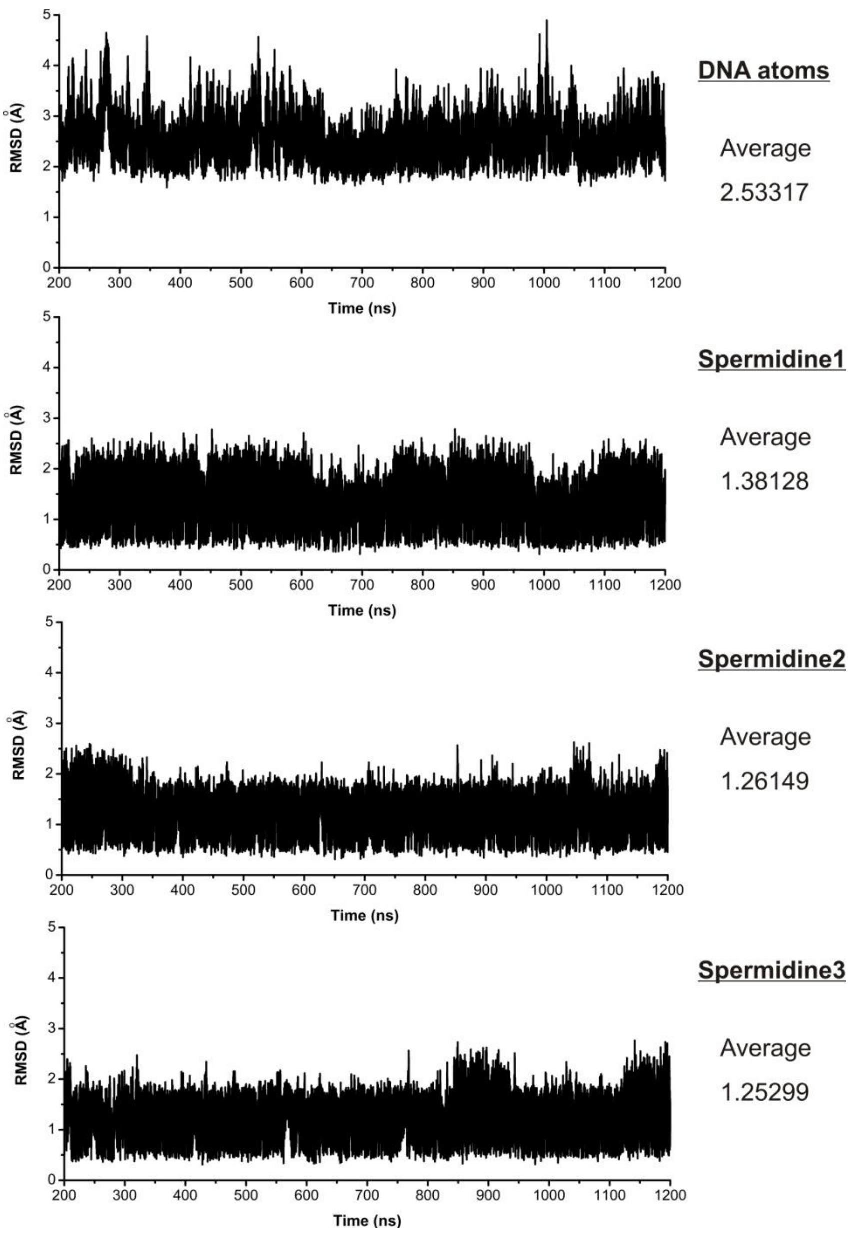

Figure S10. The root mean square deviations (RMSD) of DNA with spermidine^{3+} molecules. CHARMM36 force filed.

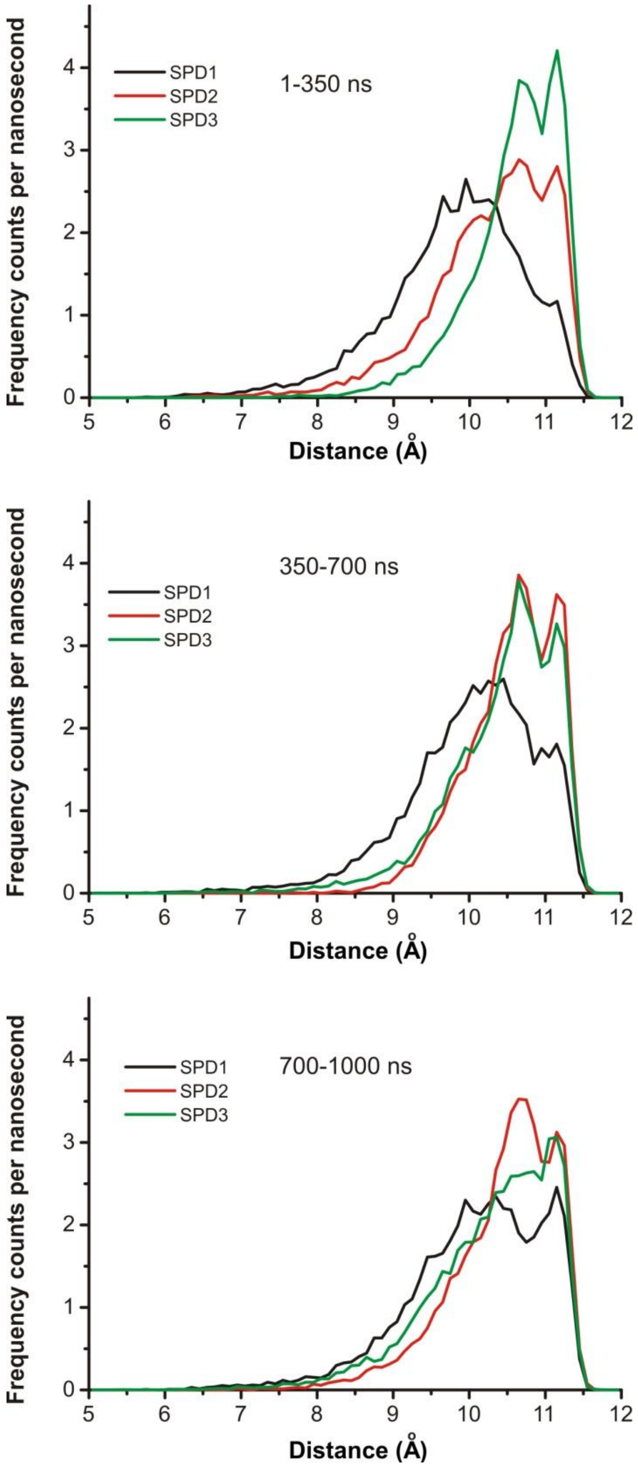

Figure S11. The distributions of the distances between the ends of spermidine molecules for different time windows of the simulation trajectory. CHARMM36 force filed.

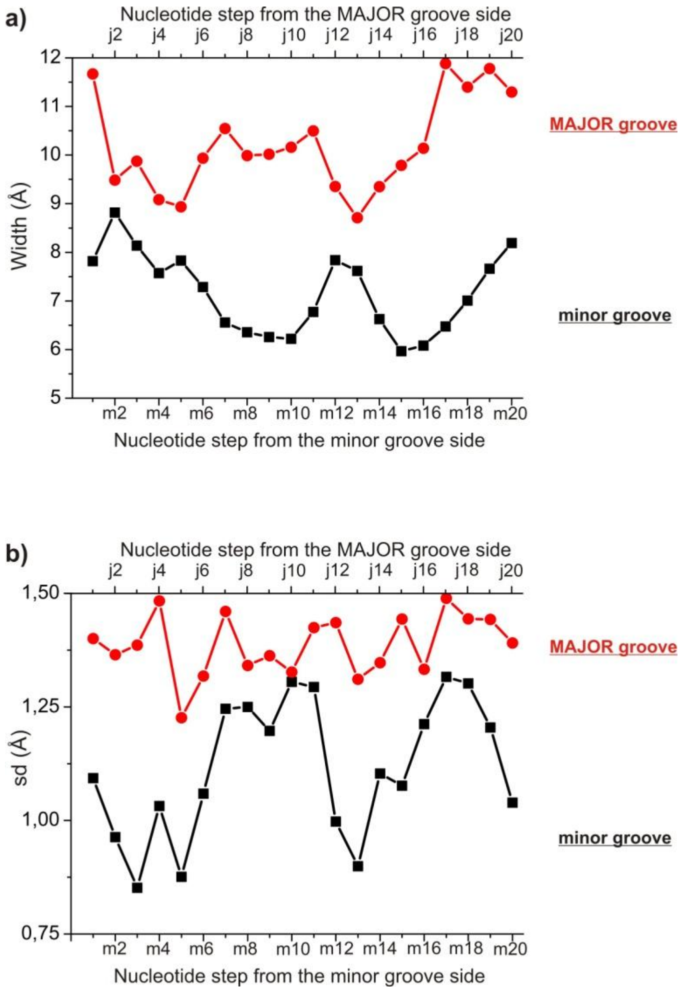

Figure S12. The dependencies of the average width (a) and standard deviations (b) of the minor and major grooves on nucleotide step in the case of DNA with spermidine^{3+}. To determine the van-der-Waals size of the groove widths the radius of the phosphates (5.8 Å) has been subtracted from the values of distances between phosphate groups. The numeration of the nucleotide steps from the minor and major groove sides are according to the Figure 1. CHARMM36 force filed.

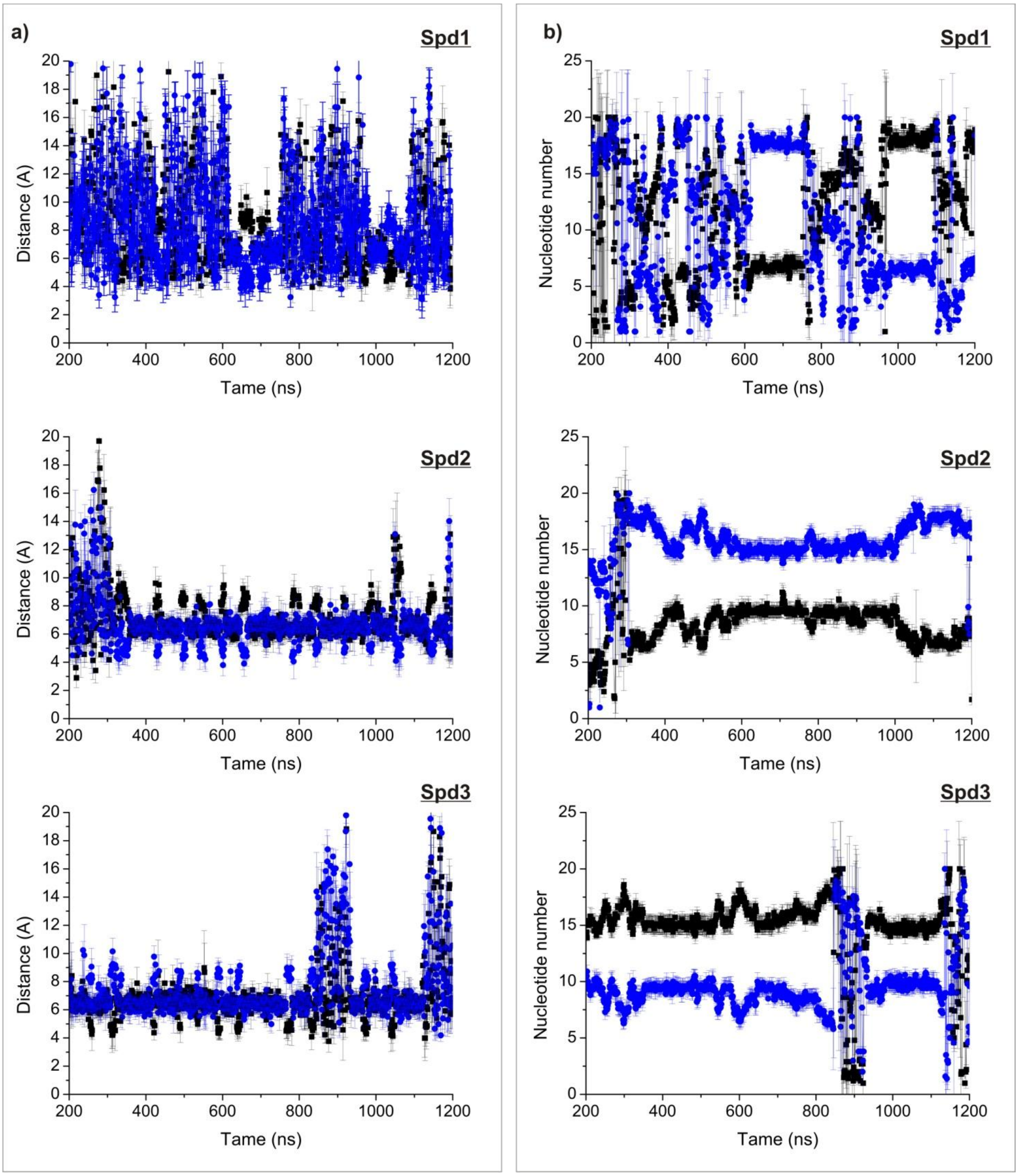

Figure S13. Localization of polyamine molecules with respect to the phosphate groups of the double helix in the system of DNA with sfpermidine. a) The time dependence of average distance from polyamine molecule to the phosphate group of the double helix (black line – chain1; blue line – chain2). The distance was averaged over all atoms of the polyamine backbone at each nanosecond of simulation tragectory. b) The time dependence of the phosphate group number which is nearest to the polyamine molecule (black line – chain1; blue line – chain2). The value of the phosphate group number was averaged over all atoms of the polyamine backbone at each nanosecond of simulation tragectory. CHARMM36 force filed.

Table S3. The residence times of spermidine molecules in different regions around DNA double helix: τ_{tot} , τ_{max} , and τ_{mean} – the total, maximal, and mean residence times of localization (in nanoseconds). The designation of regions around DNA macromolecule: mI – in the minor groove region, jI – in the major groove region, Ph– the backbone region, wX – outside region (Figure 1a). CHARMM36 force filed.

Polyamine	Localization of polyamine molecule around DNA double helix											
	mI-region			jI- region			Ph- region			wX-region		
	τ_{tot}	τ_{max}	τ_{mean}	τ_{tot}	τ_{max}	τ_{mean}	τ_{tot}	τ_{max}	τ_{mean}	τ_{tot}	τ_{max}	τ_{mean}
Spd1	277.2	44.8	2.4	87.8	3.0	0.1	28.2	0.6	0.0	606.8	22.2	1.6
Spd2	711.8	65.0	17.1	15.0	2.8	0.0	20.2	0.4	0.0	253.0	19.8	1,0
Spd3	744.6	67.4	17.0	19.8	5.6	0.0	16.0	0.4	0.0	219.6	13.0	0.6
Aver.	577.9	59.1	12.2	40.9	3.8	0.0	21.5	0.5	0.0	359.8	18.3	1.1
